# Risk factors for cognitive impairment in patients undergoing hemodialysis: an umbrella review and meta-analysis

**DOI:** 10.1080/0886022X.2026.2741985

**Published:** 2026-11-03

**Authors:** Jun Liu, Jing-Fang Wan, Jia Chen, Wei-Wei Zhang, Cai-Xia Peng, Ya-Ni He, Ke-Hong Chen

**Affiliations:** a Department of Nephrology, Daping hospital, Army Medical Center of PLA, Chongqing, China; b Chongqing Key Laboratory of Precision Diagnosis and Treatment for Kidney Diseases, Chongqing, China

**Keywords:** Risk factor, cognitive impairment, hemodialysis, meta-analysis, umbrella meta-analysis

## Abstract

Cognitive impairment is a common complication in patients undergoing maintenance hemodialysis. This umbrella review and meta-analysis systematically evaluated the factors associated with cognitive impairment and, separately, cognitive frailty in adults receiving hemodialysis. PubMed, Web of Science, Embase, and the Cochrane Library were searched from inception to January 2026. Five meta-analyses involving 97 associations were included; three evaluated cognitive impairment and two evaluated cognitive frailty. For cognitive impairment, older age, female sex, diabetes, cerebrovascular disease, multiple comorbidities, impaired physical performance, lower hemoglobin levels, and inflammatory markers were associated with increased risk, whereas higher educational attainment was associated with reduced risk (OR = 0.21, 95% CI: 0.13–0.34). For cognitive frailty, older age, female sex, depression (OR = 4.66, 95% CI: 2.98–7.27), stroke history (OR = 2.94, 95% CI: 1.72–5.03), comorbidities, malnutrition, longer dialysis duration (OR = 2.30, 95% CI: 1.49–3.53), low-flux hemodialysis (OR = 4.16, 95% CI: 1.97–8.79), and interdialytic hypotension (OR = 2.42, 95% CI: 1.53–4.21) were associated with increased risk, whereas higher education and serum albumin were protective. Only two associations had moderate-certainty evidence; all others were rated low or very low. Cognitive impairment and cognitive frailty are associated with multiple demographic, clinical, and dialysis-related factors but represent distinct outcomes that should be interpreted separately. Further high-quality prospective studies are warranted.

## 1. Introduction

A growing number of patients with chronic kidney disease (CKD) progress to end-stage renal disease (ESRD), for which maintenance hemodialysis (HD) is commonly required. In addition to cardiovascular and metabolic complications, cognitive impairment (CI) has emerged as a prevalent and clinically significant comorbidity in this population. Approximately 40–70% of patients undergoing HD exhibit measurable cognitive deficits, most commonly involving executive function and memory [1,2]. Patients undergoing dialysis face a substantially higher risk of both mild cognitive impairment and dementia. In this population, CI is independently linked to the higher rates of hospitalization and mortality [2,3]. Neuroimaging studies have reported progressive gray matter atrophy and alterations in cerebral microstructure in patients receiving HD, changes that are associated with declining cognitive performance [4,5]. Although common and clinically important, cognitive impairment in patients receiving HD often remains underrecognized in routine nephrology practice. This under recognition is partly attributable to symptom overlap with depression, fatigue, and frailty, as well as the lack of standardized screening approaches tailored to dialysis populations [6].

In patients receiving HD, cognitive impairment develops through multiple mechanisms involving both traditional vascular risk factors and dialysis-related pathophysiological processes. Cerebral small vessel disease and subsequent cognitive decline may arise from vascular comorbidities such as hypertension, diabetes, and prior stroke [7,8]. Neuronal injury and brain atrophy have also been linked to CKD-related factors such as accumulation of uremic toxins, anemia, electrolyte disturbances, systemic inflammation, and abnormalities in mineral metabolism [9,10]. Recurrent cerebral hypoperfusion and subsequent structural brain injury may occur due to hemodynamic instability during HD, particularly episodes of intradialytic hypotension [11]. Cognitive vulnerability has also been linked to modifiable lifestyle and psychosocial factors, including sedentary behavior, depression, malnutrition, and lower educational attainment [12,13]. Numerous cross-sectional and cohort studies have explored potential risk factors; however, wide variation in the magnitude and consistency of reported associations makes it difficult to identify the most reliable and clinically meaningful determinants. Moreover, many of these factors have been evaluated separately in individual studies, making it unclear which associations are supported by more consistent and higher-certainty evidence when considered together.

Several meta-analyses have examined specific dimensions of cognitive dysfunction in dialysis populations, including cognitive frailty, executive dysfunction, and deficits in particular cognitive domains [14,15]. Cognitive frailty is a multidimensional construct characterized by the coexistence of cognitive impairment and physical frailty and should not be considered interchangeable with cognitive impairment alone. Therefore, findings from studies evaluating cognitive frailty require separate interpretation from those focusing specifically on cognitive impairment. Existing syntheses have generally focused on the selected cognitive outcomes, isolated risk factors, or specific subgroups, and have not systematically compared the credibility of evidence across the broad range of potential determinants. Most studies offer only partial syntheses of the available evidence, and few integrate the full range of determinants associated with cognitive impairment in patients undergoing HD. In particular, functional factors such as gait speed, grip strength, falls, and activities of daily living (ADL) may represent components or correlates of physical frailty and cognitive frailty rather than universal risk factors for cognitive impairment. Therefore, these factors should be interpreted according to the specific outcome definitions used in individual meta-analyses. Therefore, the key unresolved issue is not whether common risk factors have been reported, but which of these reported associations are sufficiently robust, consistent, and clinically informative to support risk stratification and targeted intervention. Despite the growing burden of cognitive dysfunction and its substantial impact on prognosis and quality of life, the overall strength and credibility of the available evidence have not been systematically evaluated across studies, highlighting the need for an umbrella review with meta-analysis. Unlike previous reviews that summarized limited aspects of cognitive dysfunction in dialysis populations, this umbrella review integrates evidence across multiple categories of determinants, quantifies pooled associations where possible, and grades the credibility and certainty of the evidence. By evaluating the consistency and certainty of evidence across different factors, this study aims to distinguish more robust associations from those supported by limited evidence, rather than simply summarizing statistically significant findings from individual meta-analyses. Importantly, cognitive impairment was considered the primary outcome of interest in this review, whereas evidence from cognitive frailty studies was analyzed as a separate cognitive-related outcome according to the original definitions reported by the included meta-analyses. This study provides a comprehensive synthesis of the evidence on factors associated with cognitive impairment in patients undergoing HD, estimates pooled associations, and evaluates evidence certainty to guide targeted prevention and clinical management.

## 2. Methods and analysis

### 2.1. Design and registration

This umbrella review followed the PRISMA guidelines [16] and incorporated comprehensive literature searches, systematic data extraction, and quantitative synthesis of meta-analyses examining risk factors for cognitive impairment in patients undergoing HD. The methodological framework was guided by the Joanna Briggs Institute Manual for Evidence Synthesis [17] and the Cochrane Handbook for Systematic Reviews [18]. The study protocol was prospectively registered in PROSPERO (Registered ID: CRD420261346414).

### 2.2. Eligibility criteria

This study included published meta-analyses that assessed risk factors for cognitive impairment in patients receiving HD. Eligible studies examined demographic, clinical, laboratory, or treatment-related factors linked to cognitive impairment, cognitive dysfunction, cognitive decline, or cognitive frailty among patients receiving maintenance HD, and only articles published in English were included. Because cognitive frailty represents a multidimensional construct involving both cognitive impairment and physical frailty, studies focusing on cognitive frailty were considered separately as a related cognitive outcome rather than being regarded as equivalent to cognitive impairment. The outcomes from cognitive frailty studies were therefore interpreted according to their original definitions. Studies focusing on non-dialysis CKD populations, those restricted to peritoneal dialysis, animal experiments, narrative reviews, conference abstracts, or publications without quantitative synthesis were excluded.

#### 2.2.1. Population

Patients receive maintenance HD.

#### 2.2.2. Exposure

Included meta-analyses reported at least one factor related to cognitive impairment in patients undergoing HD, with associations expressed as odds ratios (ORs), relative risks (RRs), or hazard ratios (HRs) accompanied by corresponding 95% confidence intervals (CIs). For studies evaluating cognitive frailty, extracted associations were analyzed and reported separately from those evaluating cognitive impairment to avoid conflating these distinct clinical constructs.

#### 2.2.3. Study designs

Only meta-analyses evaluating factors associated with cognitive impairment in patients undergoing HD across different clinical settings were included, and eligible reviews were required to clearly report their search strategies, predefined inclusion and exclusion criteria, quality assessment methods, and statistical approaches used for data synthesis. Meta-analyses investigating cognitive frailty were eligible when cognitive frailty was explicitly defined according to the original study criteria and when the reported risk factors could be distinguished from those for cognitive impairment alone.

### 2.3. Information sources

Meta-analyses evaluating factors associated with cognitive impairment in patients undergoing HD were identified through a comprehensive search of PubMed, Web of Science, Embase, and the Cochrane Database from inception to 8 January 2026. Search terms included combinations of ‘hemodialysis’, ‘cognitive impairment’, ‘risk factors’, ‘meta-analysis’ and related MeSH terms, with no language or publication date restrictions. Two independent reviewers (JL and JFW) screened titles, abstracts and full texts, resolving discrepancies through consensus or consultation with a third reviewer. Eligible studies were required to report ORs or standardized mean differences (SMD) with 95% CIs for at least one modifiable or non-modifiable factor linked to cognitive decline.

### 2.4. Search strategy

MeSH terms, keywords, and text related to factors were screened from four literature databases, such as PubMed, Web of science, Embase, and Cochrane. The searching strategy in PubMed was as follows: (((((((((((((((Renal Dialysis[MeSH Terms]) OR (Dialysis[MeSH Terms])) OR (Renal Dialysis[Title/Abstract])) OR (Dialyses, Renal[Title/Abstract])) OR (Renal Dialyses[Title/Abstract])) OR (Dialysis, Renal[Title/Abstract])) OR (Hemodialysis[Title/Abstract])) OR (Hemodialyses[Title/Abstract])) OR (Dialysis, Extracorporeal[Title/Abstract])) OR (Dialyses, Extracorporeal[Title/Abstract])) OR (Extracorporeal Dialyses[Title/Abstract])) OR (Extracorporeal Dialysis[Title/Abstract])) OR (Dialysis[Title/Abstract])) OR (Dialyses[Title/Abstract])) AND (((((((((((((((((((((((((((Cognitive Dysfunction[MeSH Terms]) OR (Cognitive Dysfunction[Title/Abstract])) OR (Cognitive Dysfunctions[Title/Abstract])) OR (Dysfunction, Cognitive[Title/Abstract])) OR (Dysfunctions, Cognitive[Title/Abstract])) OR (Cognitive Disorder[Title/Abstract])) OR (Cognitive Disorders[Title/Abstract])) OR (Disorder, Cognitive[Title/Abstract])) OR (Disorders, Cognitive[Title/Abstract])) OR (Cognitive Impairments[Title/Abstract])) OR (Cognitive Impairment[Title/Abstract])) OR (Impairment, Cognitive[Title/Abstract])) OR (Impairments, Cognitive[Title/Abstract])) OR (Mild Cognitive Impairment[Title/Abstract])) OR (Cognitive Impairment, Mild[Title/Abstract])) OR (Cognitive Impairments, Mild[Title/Abstract])) OR (Impairment, Mild Cognitive[Title/Abstract])) OR (Impairments, Mild Cognitive[Title/Abstract])) OR (Mild Cognitive Impairments[Title/Abstract])) OR (Cognitive Decline[Title/Abstract])) OR (Cognitive Declines[Title/Abstract])) OR (Decline, Cognitive[Title/Abstract])) OR (Declines, Cognitive[Title/Abstract])) OR (Mental Deterioration[Title/Abstract])) OR (Deterioration, Mental[Title/Abstract])) OR (Deteriorations, Mental[Title/Abstract])) OR (Mental Deteriorations[Title/Abstract]))) AND ((meta-analysis[Title/Abstract]) OR (Systematic review[Title/Abstract])). The details of searching strategy were depicted in Table S1.

### 2.5. Study selection

Retrieved records were screened using EndNote 20, and duplicate entries were removed. Titles and abstracts were independently assessed by two reviewers to identify eligible meta-analyses on risk factors for cognitive impairment in patients undergoing hemodialysis. Disagreements were resolved through discussion with a third researcher. The study selection process is illustrated in Figure 1.

**Figure 1.**
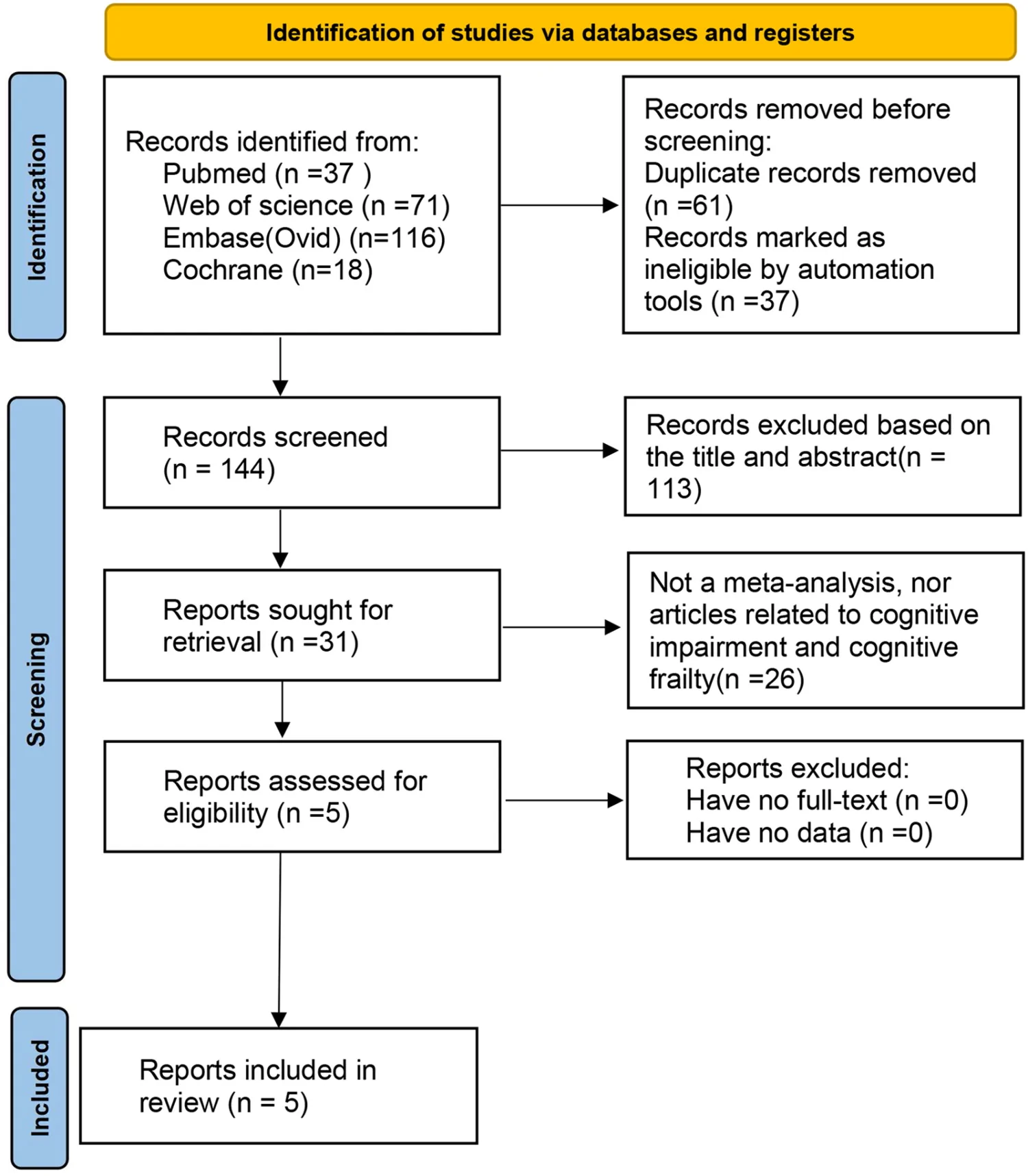
Flowchart of literature screening process.

### 2.6. Assessment of methodological quality

Methodological quality was assessed using the AMSTAR-2 tool [19], with domain-level judgments and overall confidence ratings (high, moderate, low, or critically low) applied according to the AMSTAR-2 guidance rather than summed scores. Evidence certainty was graded as high, moderate, low, or very low according to the GRADE framework [20]. AMSTAR-2 was used to evaluate the methodological quality of included systematic reviews, whereas GRADE was used to assess the certainty of individual associations. The strength of evidence was categorized as Class I (convincing), Class II (highly suggestive), Class III (suggestive), Class IV (weak), and NS (non-significant) (Table 1). AMSTAR-2 was used to evaluate the methodological quality of included systematic reviews, whereas GRADE was used to assess the certainty of individual associations. The evidence classification was performed based on the statistical results reported in the included meta-analyses. Specifically, the pooled effect estimates, *P* values, sample sizes, heterogeneity (I^2^), prediction intervals, and assessments of small-study effects or excess significance bias (when available in the original meta-analyses) were extracted and used to determine the evidence category according to Table 1 criteria. No additional meta-analyses were performed for evidence grading. Associations supported by single meta-analyses or very low-certainty evidence were interpreted cautiously.

**Table 1.** Evidence categories criteria.

Evidence class	Description
Class I: convincing evidence	>1000 cases (or >0,^20^ participants for continuous outcomes), statistical significance at *P* < 10^–6^ (random-effects), no evidence of small-study effects and excess significance bias; 95% prediction interval excluded the null, no large heterogeneity (I2 < 50%)
Class II: highly suggestive evidence	>1000 cases (or >0,^20^ participants for continuous outcomes), statistical significance at *P* < 10^–6^ (random-effects) and largest study with 95% CI excluding the null value
Class III: suggestive evidence	>1000 cases (or >20,000 participants for continuous outcomes) and statistical significance at *P* < 0.001
Class IV: weak evidence	The remaining significant associations with *P* < 0.05
NS: non-significant	*P* > 0.05

### 2.7. Data extraction

Two reviewers (JL and JW) independently extracted author name, publication year, evaluated risk factors, sample size, study design, and pooled effect estimates with corresponding 95% CIs. Effect estimates for dichotomous outcomes (RRs, ORs, and HRs) and continuous outcomes (SMDs, MDs, and correlation coefficients) were extracted as reported in the original meta-analyses. Heterogeneity (I^2^ statistics and Cochran’s Q test) and publication bias (Egger’s test) were also recorded when available. Different effect measures were not transformed or statistically pooled because they represent distinct outcome types and were interpreted within their original statistical frameworks.

### 2.8. Data summary

For associations between identified risk factors and cognitive impairment in patients undergoing hemodialysis, the pooled effect estimates (RRs, ORs, HRs, SMDs, or other effect measures) with corresponding 95% CIs were extracted from the included meta-analyses. Heterogeneity was assessed based on the reported Cochran’s Q test and I^2^ statistic. Small-study effects were extracted when reported, and sensitivity analyses and dose–response relationships were summarized when available. A *P* value <0.10 was considered indicative of significant heterogeneity, while *p* < 0.05 was considered statistically significant for other analyses.

The overlap among primary studies included in the eligible meta-analyses was assessed using the corrected covered area (CCA) method. A citation matrix was constructed, and the degree of overlap was categorized according to the established thresholds.

The strength and credibility of associations were summarized using evidence heatmaps and radar plots based on effect direction, statistical significance, evidence class, heterogeneity, and prediction interval support. Visualizations were generated using R packages (pheatmap and fmsb).

## 3. Results

### 3.1. Characteristics of meta-analyses

A total of 242 records were identified. After removal of 61 duplicates, 181 articles remained for screening. Following title and abstract screening, 37 studies were excluded for not meeting the inclusion criteria. Finally, 5 meta-analyses [14,15,21–23] were included in the umbrella review.

Across the 5 meta-analyses, 97 associations between potential risk factors and cognitive impairment in patients undergoing hemodialysis were identified. Of these, 55 associations were statistically significant, whereas 48 were non-significant. According to predefined criteria, the strength of evidence was classified as Class II, Class III, Class IV, or non-significant (NS) (Figure 2).

**Figure 2.**
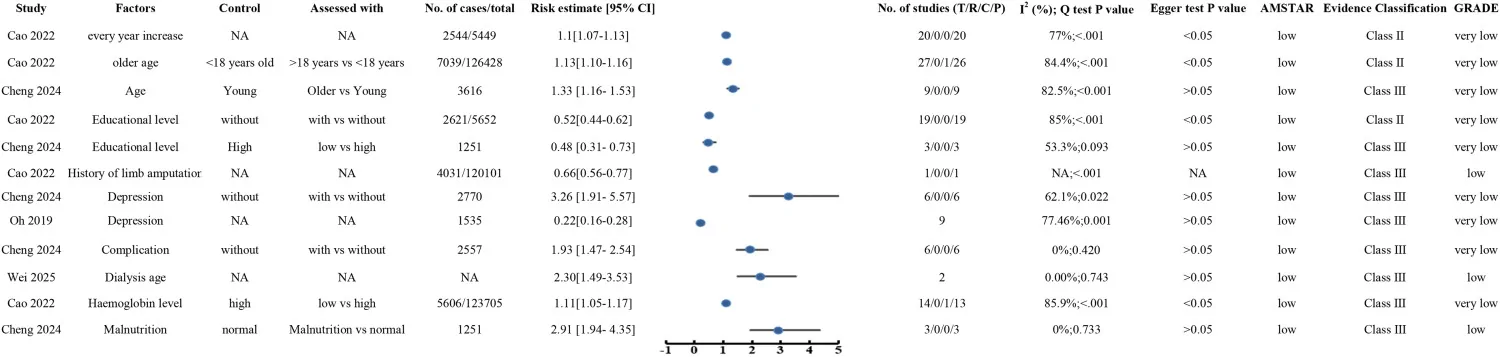
Forest plot showing pooled associations between risk factors and cognitive impairment according to evidence classification (Classes II and III evidence).

Among the included meta-analyses, three evaluated factors associated with cognitive impairment, whereas two focused on cognitive frailty. Cognitive impairment refers to a decline in cognitive function affecting one or more cognitive domains, such as memory, attention, and executive function, and is commonly assessed using validated cognitive screening tools. Cognitive frailty is a related but distinct clinical construct characterized by the coexistence of physical frailty and cognitive impairment without the established dementia. Therefore, cognitive frailty incorporates cognitive impairment as one of its components but additionally reflects physical vulnerability, and the two concepts should not be considered interchangeable. In this umbrella review, evidence from cognitive impairment and cognitive frailty meta-analyses was interpreted according to their respective outcome definitions.

### 3.2. Demographic and socioeconomic factors

#### 3.2.1. Age

All five included meta-analyses evaluated the association between age and cognitive impairment and cognitive frailty in patients undergoing hemodialysis. For cognitive impairment, Cao et al. [21] reported that each 1-year increase in age was significantly associated with increased odds cognitive impairment (OR = 1.1; 95% CI: 1.07–1.13). When age was analyzed per 10-year increment, a significant association was observed between older age and cognitive impairment (OR = 1.51; 95% CI: 1.08–2.11). Comparisons between older and younger patients also indicated a significantly higher risk among older individuals (OR = 1.13; 95% CI: 1.10–1.16). In the study by Oh et al. [23], the pooled point estimate for the association between age and cognitive impairment was 0.07 (95% CI: 0.01–0.13), indicating a weak but statistically significant positive correlation. Liu et al. [22] further showed that patients with cognitive impairment were significantly older than those with normal cognitive function (SMD = 0.49; 95% CI: 0.31–0.68).

For cognitive frailty, Cheng et al. [14] reported that older adults had a 1.33-fold higher risk than younger adults (OR = 1.33; 95% CI: 1.16–1.53). Wei et al. [15] also conducted age-stratified analyses and reported that patients aged ≥60 years and ≥75 years had higher risks of cognitive frailty than other age groups, with pooled ORs of 1.09 (95% CI: 1.06–1.12) and 3.51 (95% CI: 2.34–5.27), respectively.

Overall, increasing age appears consistently associated with a higher risk of cognitive impairment and cognitive frailty in patients undergoing hemodialysis. However, the primary studies included in these meta-analyses were predominantly observational; therefore, additional high-quality prospective studies are needed to strengthen causal inference (Table 2).

**Table 2.** Basic information of the literature.

Study	Factors	Total included MA	Factors	Outcomes	Control	Assessed with	Included MA	No. of cases/total	MA metric	Estimates [95% CI]	No. of studies(T/R/C/P)	Effects model	I^2^; Q test P value	Egger test P value	Evidence Classification	GRADE
**Demographic and socioeconomic factors**																
***Age***																
Cao 2022[21]	every year increase	20	every year increase	risk factor	NA	NA	Cao 2022	2544/5449	OR	1.1[1.07-1.13]	20/0/0/20	Random	77%;<.001	<0.05	Class II	very low
Cao 2022[21]	every 10-year increase	4	every 10-year increase	risk factor	NA	NA	Cao 2022	4695/122239	OR	1.51[1.08-2.11]	4/0/1/3	Random	95.1%;<.001	<0.05	Class IV	very low
Cao 2022[21]	older age	27	older age	risk factor	<18 years old	>18 years vs <18 years	Cao 2022	7039/126428	OR	1.13[1.10-1.16]	27/0/1/26	Random	84.4%;<.001	<0.05	Class II	very low
Cheng 2024[26]	Age	9	Age	risk factor	Young	Older vs Young	Cheng 2024	3616	OR	1.33 [1.16- 1.53]	9/0/0/9	Random	82.5%;<0.001	>0.05	Class III	very low
Liu 2023[22]	Age	9	Age	risk factor	Young	Older vs Young	Liu 2023	NA	SMD	0.49[0.31-0.68]	9/0/NA/NA	Random	84.2%;0.001	>0.05	Class IV	very low
Oh 2019[23]	Age	7	Age	risk factor	NA	NA	Oh 2019	1186	Point estimate	0.07[0.01-0.13]	7	Fixed	22.2%;0.235	>0.05	Class IV	very low
Wei 2025[15]	Age >60	2	Age >60	risk factor	Age ≤ 60	Age >60 vs Age ≤ 60	Wei 2025	NA	OR	1.09[1.06-1.12]	2	Fixed	10.0%;0.292	>0.05	Class IV	very low
Wei 2025[15]	Age >75	5	Age >75	risk factor	Age ≤ 75	Age >75 vs Age ≤ 75	Wei 2025	NA	OR	3.51[2.34-5.27]	5	Fixed	0.00%;0.898	>0.05	Class IV	very low
***Sex***																
Cao 2022[21]	Female	8	Female	risk factor	male	Female vs male	Cao 2022	4708/122382	OR	1.57[1.16-2.14]	8/0/1/7	Random	54.3%;.032	>0.05	Class IV	very low
Liu 2023[22]	Female	10	Female	risk factor	male	Female vs male	Liu 2023	NA/5535	RR	1.21[1.04-1.41]	10/0/NA/NA	Random	72.4%;0.001	>0.05	Class IV	very low
Oh 2019[23]	Gender	9	Gender	risk factor	NA	NA	Oh 2019	2018	Point estimate	0.22[0.13-0.33]	9	Random	87.91%;<.001	>0.05	Class IV	very low
Wei 2025[15]	Female	2	Female	risk factor	male	Female vs male	Wei 2025	NA	OR	2.11[1.39-3.20]	2	Fixed	0.00%;0.423	>0.05	Class IV	low
***Education***																
Cao 2022[21]	Educational level	19	Educational level	risk factor	without	with vs without	Cao 2022	2621/5652	OR	0.52[0.44-0.62]	19/0/0/19	Random	85%;<.001	<0.05	Class II	very low
Cao 2022[21]	Education (every year increase)	11	Education (every year increase)	risk factor	NA	NA	Cao 2022	1005/1978	OR	0.66[0.57-0.77]	11/0/0/11	Random	77.2%;<.001	<0.05	Class IV	very low
Cao 2022[21]	Education (≥12 years)	7	Education (≥12 years)	risk factor	<12 years	≥12 years vs <12 years	Cao 2022	1202/2057	OR	0.21[0.13-0.34]	7/0/0/7	Random	51.5%;.054	<0.05	Class IV	very low
Cheng 2024[26]	Educational level	3	Educational level	risk factor	High	low vs high	Cheng 2024	1251	OR	0.48 [0.31- 0.73]	3/0/0/3	Random	53.3%;0.093	>0.05	Class III	very low
Oh 2019[23]	Education	9	Education	risk factor	NA	NA	Oh 2019	1322	Point estimate	0.03[-0.18-0.24]	9	Random	87.04%;<.001	>0.05	NS	very low
Wei 2025[15]	Educational Level	2	Educational Level	risk factor	NA	NA	Wei 2025	NA	OR	0.33[0.22-0.51]	2	Fixed	0.00%;0.856	>0.05	Class IV	low
***Social Factors***																
Cao 2022[21]	Primary language not English	1	Primary language not English	risk factor	English	Primary language not English vs English	Cao 2022	171/383	OR	2.69[1.35-5.31]	1/0/0/1	Random	NA;—	NA	Class IV	low
Cao 2022[21]	Place of residence: hospitals	1	Place of residence: hospitals	risk factor	Other places	Hospital vs Other places	Cao 2022	4031/120101	OR	5.32[4.86-5.84]	1/0/1/0	Random	NA;<.001	NA	Class IV	moderate
Cheng 2024[26]	Social support	2	Social support	risk factor	without	with vs without	Cheng 2024	799	OR	0.65 [0.37- 1.17]	2/0/0/2	Random	88.9%;0.003	>0.05	NS	very low
**Lifestyle & Behavioral Factors**																
Cao 2022[21]	Body mass index	3	Body mass index	risk factor	NA	NA	Cao 2022	4499/121849	OR	0.98[0.93-1.04]	3/0/1/2	Random	74.6%;.020	>0.05	NS	very low
Cao 2022[21]	Sleep quality	3	Sleep quality	risk factor	good sleep quality	poor vs good sleep auality	Cao 2022	250/406	OR	2.91[0.91-9.32]	3/0/0/3	Random	89%;<.001	>0.05	NS	very low
Oh 2019[23]	Sleep difficulties	3	Sleep difficulties	risk factor	NA	NA	Oh 2019	370	Point estimate	0.15[0.05-0.25]	3	Fixed	0%;0.822	>0.05	Class IV	very low
Cheng 2024[26]	Waistline	2	Waistline	risk factor	NA	NA	Cheng 2024	792	OR	1.05 [1.03- 1.08]	2/0/0/2	Fixed	0%;0.460	>0.05	Class IV	very low
Wei 2025[15]	Waist	2	Waist	risk factor	NA	NA	Wei 2025	NA	OR	1.05[1.03-1.08]	2	Fixed	0.00%;0.46	>0.05	Class IV	very low
Wei 2025[15]	Calf circumference	2	Calf circumference	risk factor	NA	NA	Wei 2025	NA	OR	0.93[0.88-0.97]	2	Fixed	0.00%;0.495	>0.05	Class IV	very low
Liu 2023[22]	Smoking use	5	Smoking use	risk factor	without	with vs without	Liu 2023	NA	RR	0.74[0.52-1.07]	5/0/NA/NA	Random	73.2%;0.005	>0.05	NS	very low
Liu 2023[22]	Alcohol use	3	Alcohol use	risk factor	without	with vs without	Liu 2023	NA	RR	0.96[0.77-1.2]	3/0/NA/NA	Fixed	0%;0.948	>0.05	NS	very low
Wei 2025[15]	Exercise	2	Exercise	risk factor	NA	NA	Wei 2025	NA	OR	1.79[0.10-30.87]	2	Random	83.50%;0.014	>0.05	NS	very low
**Functional Status & Physical Performance**																
Cao 2022[21]	Slow gait	1	Slow gait	risk factor	NA	NA	Cao 2022	134/467	OR	2.15[1.05-4.42]	1/0/0/1	Random	NA;.037	NA	Class IV	low
Cao 2022[21]	Low grip strength	1	Low grip strength	risk factor	NA	NA	Cao 2022	134/467	OR	3.19[1.55-6.55]	1/0/0/1	Random	NA;.002	NA	Class IV	low
Cao 2022[21]	Activities of daily living	1	ADL	risk factor	NA	NA	Cao 2022	4031/120101	OR	1.13[1.05-1.22]	1/0/0/1	Random	NA;.001	NA	Class IV	low
Oh 2019[23]	ADL	3	ADL	risk factor	NA	NA	Oh 2019	339	Point estimate	0.35[0.27-0.42]	3	Fixed	38.89%;0.195	>0.05	Class IV	very low
Oh 2019[23]	Pain	3	Pain	risk factor	NA	NA	Oh 2019	233	Point estimate	0.13[0.01-0.25]	3	Fixed	29.95%;0.107	>0.05	NS	very low
Cao 2022[21]	History of limb amputation	1	History of limb amputation	risk factor	NA	NA	Cao 2022	4031/120101	OR	0.66[0.56-0.77]	1/0/0/1	Random	NA;<.001	NA	Class III	low
Cao 2022[21]	History of hip fracture	1	History of hip fracture	risk factor	NA	NA	Cao 2022	4031/120101	OR	2.38[2.10-2.71]	1/0/0/1	Random	NA;<.001	NA	Class IV	moderate
Wei 2025[15]	Fall history	2	Fall history	risk factor	without	with vs without	Wei 2025	NA	OR	3.41[1.98-5.88]	2	Fixed	0.00%;0.859	>0.05	Class IV	low
**Psychological & Mental Health Factors**																
Cao 2022[21]	Depression status	1	Depression status	risk factor	normal	depression vs normal	Cao 2022	27/108	OR	1.05[1.01-1.10]	1/0/0/1	Random	NA;0.015	NA	Class IV	very low
Cheng 2024[26]	Depression	6	Depression	risk factor	without	with vs without	Cheng 2024	2770	OR	3.26 [1.91- 5.57]	6/0/0/6	Random	62.1%;0.022	>0.05	Class III	very low
Oh 2019[23]	Depression	9	Depression	risk factor	NA	NA	Oh 2019	1535	Point estimate	0.22[0.16-0.28]	9	Random	77.46%;0.001	>0.05	Class III	very low
Wei 2025[15]	Depression(HADS)	3	Depression(HADS)	risk factor	without	with vs without	Wei 2025	NA	OR	4.66[2.98-7.27]	3	Fixed	0.00%;0.338	>0.05	Class IV	low
Wei 2025[15]	Depression	3	Depression	risk factor	without	with vs without	Wei 2025	NA	OR	0.59[0.01-26.28]	3	Random	90.00%;0.002	>0.05	NS	very low
Wei 2025[15]	Depression(SDS)	2	Depression(SDS)	risk factor	without	with vs without	Wei 2025	NA	OR	1.50[0.54-4.22]	2	Random	76.50%;0.039	>0.05	NS	very low
Wei 2025[15]	Geriatric Depression Scale score(GDS-15)	2	Geriatric Depression Scale score(GDS-15)	risk factor	NA	NA	Wei 2025	NA	OR	1.27[0.95-1.70]	2	Random	14.30%;0.28	>0.05	NS	very low
Cao 2022[21]	Hope level	1	Hope level	risk factor	NA	NA	Cao 2022	84/120	OR	0.01[0-0.16]	1/0/0/1	Random	NA;.018	NA	Class IV	low
Cao 2022[21]	Psychological status	1	Psychological status	risk factor	NA	NA	Cao 2022	79/138	OR	6.01[1.91-17.32]	1/0/0/1	Random	NA;<.05	NA	Class IV	low
**Cardiovascular & Hemodynamic Factors**																
***Disease***																
Cao 2022[21]	Hypertension	4	Hypertension	risk factor	without	with vs without	Cao 2022	257/515	OR	4.36[1.56-12.15]	4/0/0/4	Random	86.3%;<.001	>0.05	Class IV	very low
Liu 2023[22]	Hypertension	6	Hypertension	risk factor	without	with vs without	Liu 2023	NA	RR	1.06[0.97-1.15]	6/0/NA/NA	Fixed	0%;0.448	>0.05	NS	very low
Oh 2019[23]	Hypertension	7	Hypertension	risk factor	NA	NA	Oh 2019	1894	Point estimate	0.09[-0.07-0.24]	7	Random	91.04%;<.001	>0.05	NS	very low
Cao 2022[21]	Diabetes	14	Diabetes	risk factor	without	with vs without	Cao 2022	6016/124440	OR	2.12[1.52-2.96]	14/0/1/13	Random	86.1%;<.001	<0.05	Class IV	very low
Liu 2023[22]	Diabetes	7	Diabetes	risk factor	without	with vs without	Liu 2023	NA	RR	1.33[1.04-1.71]	7/0/NA/NA	Random	53.2%;0.046	>0.05	Class IV	very low
Oh 2019[23]	Diabetes	7	Diabetes	risk factor	NA	NA	Oh 2019	1312	Point estimate	0.03[-1.13-1.18]	7	Random	81.93%;<.001	>0.05	NS	very low
Cao 2022[21]	Heart disease	5	Heart disease	risk factor	without	with vs without	Cao 2022	4582/122072	OR	1.25[0.96-1.62]	5/0/1/4	Random	67.7%;.015	>0.05	NS	very low
Liu 2023[22]	Coronary heart disease	2	Coronary heart disease	risk factor	without	with vs without	Liu 2023	NA	RR	0.86[0.61-1.21]	2/0/NA/NA	Random	66.1%;0.086	>0.05	NS	very low
Oh 2019[23]	CVD	9	CVD	risk factor	NA	NA	Oh 2019	2293	Point estimate	0.14[-0.11-0.26]	9	Random	88.49%;<.001	>0.05	NS	very low
Cao 2022[21]	Cerebrovascular accident	4	Cerebrovascular accident	risk factor	without	with vs without	Cao 2022	1015/2505	OR	1.9[1.39-2.60]	4/0/0/4	Random	39.2%;.177	>0.05	Class IV	very low
Liu 2023[22]	Cerebrovascular disease	4	Cerebrovascular disease	risk factor	without	with vs without	Liu 2023	NA	RR	1.06[0.79-1.42]	4/0/NA/NA	Random	84.2%;0.001	>0.05	NS	very low
Cheng 2024[26]	Stroke history	3	Stroke history	risk factor	without	with vs without	Cheng 2024	763	OR	2.94 [1.72- 5.03]	3/0/0/3	Fixed	0%;0.999	>0.05	Class IV	low
Liu 2023[22]	Stroke	3	Stroke	risk factor	without	with vs without	Liu 2023	NA	RR	1.66[1.08-2.55]	3/0/NA/NA	Random	78.1%;0.01	>0.05	Class IV	very low
Oh 2019[23]	Stroke	4	Stroke	risk factor	NA	NA	Oh 2019	968	Point estimate	0.1[0.06-0.13]	4	Fixed	0%;0.767	>0.05	Class IV	very low
Wei 2025[15]	Stroke history	2	Stroke history	risk factor	without	with vs without	Wei 2025	NA	OR	2.92[1.53-5.57]	2	Fixed	0.00%;0.976	>0.05	Class IV	low
***hemodynamic indicators***																
Cao 2022[21]	SBPV	2	SBPV	risk factor	NA	NA	Cao 2022	237/330	OR	1.17[1.05-1.30]	2/0/0/2	Random	0%;.452	>0.05	Class IV	very low
Cao 2022[21]	Mean systolic blood pressure	1	Mean systolic blood pressure	risk factor	NA	NA	Cao 2023	55/105	OR	0.94[0.87-1.01]	1/0/0/1	Random	NA;.075	NA	NS	very low
Cao 2022[21]	Arterial stiffness	2	Arterial stiffness	risk factor	NA	NA	Cao 2022	225/378	OR	1.38[1.23-1.56]	2/0/0/2	Random	2%;.312	>0.05	Class IV	very low
**Comorbidity Burden**																
Cao 2022[21]	Multiple comorbid conditions	3	Multiple comorbid conditions	risk factor	without	with vs without	Cao 2022	352/631	OR	3.8[2.35-6.15]	3/0/0/3	Random	21.7%;.279	>0.05	Class IV	low
Wei 2025[15]	Comorbidities	2	Comorbidities	risk factor	without	with vs without	Wei 2025	NA	OR	2.18[1.22-3.90]	2	Fixed	41.90%;0.19	>0.05	Class IV	low
Wei 2025[15]	Complications	2	Complications	risk factor	without	with vs without	Wei 2025	NA	OR	2.13[1.34-3.40]	2	Fixed	6.40%;0.301	>0.05	Class IV	low
Cheng 2024[26]	Complication	6	Complication	risk factor	without	with vs without	Cheng 2024	2557	OR	1.93 [1.47- 2.54]	6/0/0/6	Fixed	0%;0.420	>0.05	Class III	very low
Wei 2025[15]	CCI	2	CCI	risk factor	NA	NA	Wei 2025	NA	OR	2.67[1.66-4.32]	2	Fixed	0.00%;0.996	>0.05	Class IV	low
**Kidney Disease & Dialysis-Related Factors**																
***Dialysis Parameters***																
Cao 2022[21]	Haemodialysis duration	13	Haemodialysis duration	risk factor	shorter	longer vs shorter haemodialysis duration	Cao 2022	5883/124032	OR	1[0.98-1.03]	13/0/1/12	Random	93.5%;<.001	<0.05	NS	very low
Cheng 2024[26]	Dialysis duration	4	Dialysis duration	risk factor	NA	NA	Cheng 2024	2265	OR	1.53 [0.96- 2.41]	4/0/0/4	Random	71.6%;0.014	>0.05	NS	very low
Oh 2019[23]	Duration	7	Duration	risk factor	NA	NA	Oh 2019	984	Point estimate	0[-0.06-0.06]	7	Fixed	19.87%;0.278	>0.05	NS	very low
Liu 2023[22]	Time on dialysis	6	Time on dialysis	risk factor	NA	NA	Liu 2023	NA	SMD	0.42[-0.06-0.89]	6/0/NA/NA	Random	93.8%;0.001	>0.05	NS	very low
Cao 2022[21]	Haemodialysis adequacy	7	Haemodialysis adequacy	risk factor	Insufficient	Sufficient vs Insufficient	Cao 2022	5204/122907	OR	0.68[0.46-1.01]	7/0/1/6	Random	92.3%;<.001	>0.05	NS	low
Cao 2022[21]	Haemodialysis frequency	2	Haemodialysis frequency	risk factor	NA	NA	Cao 2022	582/1841	OR	0.65[0.20-2.11]	2/0/0/2	Random	80.3%;.024	>0.05	NS	very low
Oh 2019[23]	Effects	9	Effects	risk factor	NA	NA	Oh 2019	1419	Point estimate	−0.06[-0.15 ∼ 0.03]	9	Random	79.81%;<.001	>0.05	NS	very low
Wei 2025[15]	Dialysis age	2	Dialysis age	risk factor	NA	NA	Wei 2025	NA	OR	2.30[1.49-3.53]	2	Fixed	0.00%;0.743	>0.05	Class III	low
Cao 2022[21]	Post-dialysis diastolic pressure	1	Post-dialysis diastolic pressure	risk factor	NA	NA	Cao 2022	113/219	OR	2.23[1.25-3.98]	1/0/0/1	Random	NA;.006	NA	Class IV	low
Cao 2022[21]	Excessive body weight changes before and after dialysis	1	Excessive body weight changes before and after dialysis	risk factor	normal	Excessive vs normal	Cao 2022	55/105	OR	0.05[0.01-0.42]	1/0/0/1	Random	NA;.006	NA	Class IV	low
Cao 2022[21]	Interdialysis hypotension	1	Interdialysis hypotension	risk factor	normal	low vs normal	Cao 2022	31/78	OR	2.42[1.53-4.21]	1/0/0/1	Random	NA;<.001	NA	Class IV	low
Cao 2022[21]	Nocturnal haemodialysis	1	Nocturnal haemodialysis	risk factor	other time	Nocturnal haemodialysis vs other time	Cao 2022	171/383	OR	0.41[0.17-0.87]	1/0/0/1	Random	NA;—	NA	Class IV	low
Cao 2022[21]	Low-flux haemodialysis	1	Low-flux haemodialysis	risk factor	normal	low vs normal	Cao 2022	202/315	OR	4.16[1.97-8.79]	1/0/0/1	Random	NA;<.001	NA	Class IV	low
Cao 2022[21]	Dry weight	2	Dry weight	risk factor	NA	NA	Cao 2022	56/262	OR	0.96[0.93-1.01]	2/0/0/2	Random	3.9%;.308	>0.05	NS	low
***Renal Function Markers***																
Cao 2022[21]	Creatinine	4	Creatinine	risk factor	NA	NA	Cao 2022	604/2049	OR	1[0.92-1.10]	4/0/0/4	Random	74.3%;.009	>0.05	NS	very low
Oh 2019[23]	Creatinine	3	Creatinine	risk factor	NA	NA	Oh 2019	258	Point estimate	0.09[-0.03-0.20]	3	Random	29.47%;<.001	>0.05	NS	very low
Cheng 2024[26]	Serum creatinine	3	Serum creatinine	risk factor	NA	NA	Cheng 2024	2007	OR	1.00 [0.99- 1.00]	3/0/0/3	Fixed	0%;0.404	>0.05	NS	very low
Wei 2025[15]	Serum creatinine level	3	Serum creatinine level	risk factor	NA	NA	Wei 2025	NA	OR	1.00[0.99-1.00]	3	Fixed	0.00%;0.404	>0.05	NS	very low
Oh 2019[23]	BUN	4	BUN	risk factor	NA	NA	Oh 2019	695	Point estimate	−0.02[-0.11-0.07]	4	Fixed	19.74%;0.291	>0.05	NS	very low
Cao 2022[21]	Cystatin C	1	Cystatin C	risk factor	NA	NA	Cao 2022	68/120	OR	4.08[1.78-9.33]	1/0/0/1	Random	NA;.001	NA	Class IV	low
Cao 2022[21]	Residual urine volume	1	Residual urine volume	risk factor	NA	NA	Cao 2022	38/93	OR	0.236[0.07-0.82]	1/0/0/1	Random	NA;.024	NA	Class IV	low
Cao 2022[21]	Chronic glomerulonephritis	1	Chronic glomerulonephritis	risk factor	NA	NA	Cao 2022	414/1617	OR	0.85[0.62-1.17]	1/0/0/1	Random	NA;.317	NA	NS	very low
**Metabolic, Nutritional & Inflammatory Markers**																
Cao 2022[21]	Haemoglobin level	14	Haemoglobin level	risk factor	high	low vs high	Cao 2022	5606/123705	OR	1.11[1.05-1.17]	14/0/1/13	Random	85.9%;<.001	<0.05	Class III	very low
Cheng 2024[26]	Hemoglobin	3	Hemoglobin	risk factor	NA	NA	Cheng 2024	953	OR	0.67 [0.36- 1.26]	3/0/0/3	Random	74.3%;0.020	>0.05	NS	very low
Oh 2019[23]	Hemoglobin	8	Hemoglobin	risk factor	NA	NA	Oh 2019	1328	Point estimate	0.1[0.04-0.16]	8	Fixed	24.13%;0.229	>0.05	Class IV	very low
Wei 2025[15]	Hemoglobin	3	Haemoglobin	risk factor	NA	NA	Wei 2025	NA	OR	1.00[0.38-2.64]	3	Random	87.70%;0	>0.05	NS	very low
Cao 2022[21]	Serum albumin level	5	Serum albumin level	risk factor			Cao 2022	4705/122377	OR	0.84[0.72-0.99]	4/0/1/3	Random	81.6%;<.001	>0.05	Class IV	low
Cheng 2024[26]	Serum albumin	4	Serum albumin	risk factor	NA	NA	Cheng 2024	1293	OR	0.72 [0.41- 1.27]	4/0/0/4	Random	82.9%;0.001	>0.05	NS	very low
Liu 2023[22]	Albumin	3	Albumin	risk factor	NA	NA	Liu 2023	NA	SMD	−0.2[-0.42-0.03]	3/0/NA/NA	Random	66.7%;0.05	>0.05	NS	very low
Oh 2019[23]	Albumin	6	Albumin	risk factor	NA	NA	Oh 2019	930	Point estimate	0.02[-0.03-0.07]	6	Fixed	53.96%;0.054	>0.05	NS	very low
Wei 2025[15]	Serum albumin	6	Serum albumin	risk factor	NA	NA	Wei 2025	NA	OR	0.80[0.66-0.90]	6	Random	74.90%;0.001	>0.05	Class IV	very low
Cao 2022[21]	25(OH)D	2	25(OH)D	risk factor	NA	NA	Cao 2022	121/285	OR	0.95[0.78-1.15]	2/0/0/2	Random	76.8%;.038	>0.05	NS	very low
Cao 2022[21]	CRP level	2	CRP level	risk factor	NA	NA	Cao 2022	4115/120221	OR	1.76[0.55-5.66]	2/0/1/1	Random	91.8%;<.001	>0.05	NS	low
Cao 2022[21]	Homocysteine	2	Homocysteine	risk factor	NA	NA	Cao 2022	NA	OR	1.88[0.69-5.07]	NA	Random	83.9%;.013	>0.05	NS	very low
Cao 2022[21]	Serum cholesterol	2	Serum cholesterol	risk factor	NA	NA	Cao 2022	81/239	OR	1.8[0.50-2.34]	2/0/0/2	Random	65.2%;.090	>0.05	NS	very low
Cao 2022[21]	Serum uric acid	4	Serum uric acid	risk factor	NA	NA	Cao 2022	279/564	OR	3.01[0.65-14.02]	4/0/0/4	Random	98.2%;<.001	>0.05	NS	low
Cao 2022[21]	PTH	6	PTH	risk factor	NA	NA	Cao 2022	801/1295	OR	1.002[0.999-1.006]	4/0/0/4	Random	75.5%;.001	>0.05	NS	very low
Cao 2022[21]	Sodium	1	Sodium	risk factor	NA	NA	Cao 2022	29/154	OR	0.86[0.74-1.00]	1/0/0/1	Random	NA;.05	na	NS	very low
Cao 2022[21]	Potassium	1	Potassium	risk factor	NA	NA	Cao 2022	29/154	OR	0.77[0.33-1.83]	1/0/0/1	Random	NA;.558	NA	NS	very low
Cao 2022[21]	Phosphorus	1	Phosphorus	risk factor	NA	NA	Cao 2022	414/1617	OR	0.99[0.90-1.09]	1/0/0/1	Random	NA;.785	NA	NS	very low
Cao 2022[21]	BUN	1	BUN	risk factor	NA	NA	Cao 2022	NA	OR	1.01[0.97-1.05]	NA	Random	NA;.578	NA	NS	very low
Cheng 2024[26]	Malnutrition	3	Malnutrition	risk factor	normal	Malnutrition vs normal	Cheng 2024	1251	OR	2.91 [1.94- 4.35]	3/0/0/3	Fixed	0%;0.733	>0.05	Class III	low
Wei 2025[15]	Malnutrition	3	Malnutrition	risk factor	NA	NA	Wei 2025	NA	OR	2.78[1.83-4.22]	3	Fixed	0.00%;0.601	>0.05	Class IV	low
Wei 2025[15]	Triglycerides	2	Triglycerides	risk factor	NA	NA	Wei 2025	NA	OR	4.03[1.87-8.70]	2	Fixed	0.00%;0.563	>0.05	Class IV	low
Cao 2022[21]	Erythrocyte count	1	Erythrocyte count	risk factor	NA	NA	Cao 2022	29/154	OR	1[0.98-1.01]	1/0/0/1	Random	NA;.537	NA	NS	very low
Cao 2022[21]	Fibroblast growth factor-23	1	Fibroblast growth factor-23	risk factor	NA	NA	Cao 2022	22/69	OR	0.6[0.37-0.88]	1/0/0/1	Random	NA;.019	NA	Class IV	very low
**Neurological Injury Biomarkers**																
Cao 2022[21]	Plasma T-tau	1	Plasma T-tau	risk factor	NA	NA	Cao 2022	45/87	OR	1.52[1.17-1.98]	1/0/0/1	Random	NA;.002	NA	Class IV	very low
Cao 2022[21]	Neuron-specific enolase	1	Neuron-specific enolase	risk factor	NA	NA	Cao 2022	13/30	OR	12.33[0.60-251.39]	1/0/0/1	Random	NA;.102	NA	NS	low
Cao 2022[21]	S100B	1	S100B	risk factor	NA	NA	Cao 2022	13/30	OR	11.3[1.02-124.19]	1/0/0/1	Random	NA;.047	NA	Class IV	low
Cao 2022[21]	Intracranial artery calcification	1	Intracranial artery calcification	risk factor	NA	NA	Cao 2022	22/69	OR	2.65[0.49-16.04]	1/0/0/1	Random	NA;.262	NA	NS	low
**Body Composition & Fluid Status**																
Cao 2022[21]	Lean tissue mass	1	Lean tissue mass	risk factor	NA	NA	Cao 2022	105/180	OR	5.28[1.03-27.15]	1/0/0/1	Random	NA;.046	NA	Class IV	low
Cao 2022[21]	Total body water	1	Total body water	risk factor	NA	NA	Cao 2022	105/180	OR	1.68[0.32-8.75]	1/0/0/1	Random	NA;.538	NA	NS	very low
Cao 2022[21]	Extracellular water	1	Extracellular water	risk factor	NA	NA	Cao 2022	105/180	OR	4.42[0.75-26.18]	1/0/0/1	Random	NA;.101	NA	NS	low
**Hematologic Inflammatory Ratios**																
Cao 2022[21]	Neutrophil percentage	1	Neutrophil percentage	risk factor	NA	NA	Cao 2022	38/93	OR	1.156[1.06-1.27]	1/0/0/1	Random	NA;.002	NA	Class IV	very low
Cao 2022[21]	Platelet/lymphocyte ratio	1	Platelet/lymphocyte ratio	risk factor	NA	NA	Cao 2022	970/2019	OR	2.73[1.90-3.91]	1/0/0/1	Random	NA;<.001	NA	Class IV	low
Cao 2022[21]	Neutrophils/lymphocyte ratio	1	Neutrophils/lymphocyte ratio	risk factor	NA	NA	Cao 2022	NA	OR	1.89[1.31-2.72]	NA	Random	NA;<.001	NA	Class IV	very low

ADL: Activities of Daily Living; CCI: Charlson Comorbidity Index; CVD: Cardiovascular Disease; SBPV: Systolic Blood Pressure Variability; BUN: Blood Urea Nitrogen; CRP: C-Reactive Protein; PTH: Parathyroid Hormone; 25(OH)D: 25-Hydroxyvitamin D; FGF-23: Fibroblast Growth Factor 23; HADS: Hospital Anxiety and Depression Scale; SDS: Self-Rating Depression Scale; GDS-15: 15-item Geriatric Depression Scale; S100B: S100 Calcium-Binding Protein B; PLR: Platelet-to-Lymphocyte Ratio; NLR: Neutrophil-to-Lymphocyte Ratio

The red font indicates non-significance, while the black font indicates significance.

#### 3.2.2. Sex

Four meta-analyses examined the association between sex and cognitive impairment and cognitive frailty in patients undergoing hemodialysis. Regarding sex, three meta-analyses evaluated the association between sex and cognitive impairment in patients receiving HD. Cao et al. [21] and Liu et al. [22] both reported that female patients had a higher risk of cognitive impairment compared with male patients (OR = 1.57; 95% CI: 1.16–2.14 and OR = 1.21; 95% CI: 1.04–1.41, respectively). Oh et al. [23] also identified a statistically significant association between sex and cognitive impairment, reporting a pooled estimate of 0.22 (95% CI: 0.13–0.33); however, the effect measure and reference category differed from the OR-based estimates, limiting direct comparison with the other two analyses.

When cognitive frailty was considered separately, Wei et al. [15] reported a significant association between female sex and cognitive frailty, with female patients having a higher likelihood of cognitive frailty than male patients (OR = 2.11; 95% CI: 1.39–3.20).

Overall, female sex was consistently associated with cognitive-related outcomes in patients undergoing HD, including cognitive impairment and cognitive frailty. However, interpretation remains cautious due to heterogeneity in outcome definitions and effect measures across meta-analyses (Table 2).

#### 3.2.3. Education

Four meta-analyses examined the association between educational attainment and cognitive-related outcomes (cognitive impairment and cognitive frailty) in patients undergoing hemodialysis. Cao et al. [21] reported that higher educational level was associated with a significantly lower risk of cognitive impairment than lower education (OR = 0.52; 95% CI: 0.44–0.62). Each additional year of education was associated with a reduced risk (OR = 0.66; 95% CI: 0.57–0.77). Individuals with ≥12 years of education also had a markedly lower risk than those with <12 years (OR = 0.21; 95% CI: 0.13–0.34). Oh et al. [23] also reported a statistically significant association between education and cognitive performance using a pooled point estimate (0.03; 95% CI: −0.18–0.24); however, the effect metric differed from the OR-based estimates, limiting direct comparison.

For cognitive frailty, Cheng et al. [14] reported that lower educational level was associated with a higher risk than higher education (OR = 0.48; 95% CI: 0.31–0.73). Wei et al. [15] also reported a protective association of higher educational level (OR = 0.33; 95% CI: 0.22–0.51).

Overall, higher educational attainment was consistently associated with a lower likelihood of cognitive-related outcomes, including cognitive impairment and cognitive frailty, among patients undergoing HD (Table 2).

#### 3.2.4. Social factor

Two meta-analyses examined social factors in relation to cognitive impairment and cognitive frailty among patients undergoing hemodialysis. Cao et al. [21] reported that having a primary language other than English was associated with a significantly higher risk of cognitive impairment than English speakers (OR = 2.69; 95% CI: 1.35–5.31). The same study found that residence in hospitals, compared with other places of residence, was associated with a markedly higher risk (OR = 5.32; 95% CI: 4.86–5.84).

For cognitive frailty, Cheng et al. [14] evaluated social support and reported that absence of social support was not significantly associated with cognitive impairment compared with having support (OR = 0.65; 95% CI: 0.37–1.17).

Overall, certain social determinants, particularly language background and place of residence, may be associated with an increased risk of cognitive impairment in hemodialysis populations, while evidence regarding social support and cognitive frailty remains limited and inconclusive (Table 2).

### 3.3. Lifestyle and behavioral factors

Five meta-analyses examined lifestyle and behavioral factors in relation to cognitive impairment and cognitive frailty among patients undergoing hemodialysis. For cognitive impairment, Cao et al. [21] reported that body mass index was not significantly associated with cognitive impairment (OR = 0.98; 95% CI: 0.93–1.04), whereas poor sleep quality was associated with a higher risk compared with good sleep (OR = 2.91; 95% CI: 0.91–9.32). Oh et al. [23] also identified a significant association between sleep difficulties and cognitive impairment (point estimate = 0.15; 95% CI: 0.05–0.25). Liu et al. [22] reported that smoking (RR= 0.74; 95% CI: 0.52–1.07) and alcohol consumption (RR= 0.96; 95% CI: 0.77–1.2) were not significantly associated with cognitive impairment.

For cognitive frailty, Cheng et al. [14] and Wei et al. [15] consistently reported that greater waist circumference was associated with an increased risk of cognitive frailty (OR = 1.05; 95% CI: 1.03–1.08). Wei et al. [15] further reported that limited exercise was associated with a higher risk of cognitive frailty (OR = 1.79; 95% CI: 0.10–30.87), although the estimate was accompanied by a wide confidence interval.

Overall, lifestyle and behavioral factors showed differential associations with cognitive-related outcomes among patients undergoing HD. Sleep disturbances may be associated with cognitive impairment, whereas central obesity and limited exercise were associated with cognitive frailty. Associations involving body mass index, smoking, and alcohol consumption remained uncertain (Table 2).

### 3.4. Functional status and physical performance

Three meta-analyses evaluated functional status and physical performance in relation to cognitive impairment and cognitive frailty among patients undergoing hemodialysis. For cognitive impairment, Cao et al. [21] reported that slow gait (OR = 2.15; 95% CI: 1.05–4.42), low grip strength (OR = 3.19; 95% CI: 1.55–6.55), and impaired activities of daily living (OR = 1.13; 95% CI: 1.05–1.22) were significantly associated with an increased risk of cognitive impairment. The same study reported that a history of hip fracture was associated with a higher risk (OR = 2.38; 95% CI: 2.10–2.71), whereas a history of limb amputation showed a statistically significant association in the opposite direction (OR = 0.66; 95% CI: 0.56–0.77). Oh et al. [23] identified significant associations of impaired ADL (point estimate = 0.35; 95% CI: 0.27–0.42) and not significant association in pain (point estimate = 0.13; 95% CI: 0.01–0.25) with cognitive impairment.

For cognitive frailty, Wei et al. [15] reported that a history of falls was associated with an increased risk of cognitive frailty (OR = 3.41; 95% CI: 1.98–5.88).

Overall, impaired physical performance and functional limitations were associated with cognitive impairment, whereas falls were associated with cognitive frailty when assessed as a separate cognitive-related outcome. These findings indicate that physical function may be related to cognitive-related outcomes among patients undergoing HD; however, the interpretation should consider differences in outcome definitions across meta-analyses (Table 2).

### 3.5. Psychological and mental health factors

Four meta-analyses evaluated psychological and mental health factors in relation to cognitive impairment among patients undergoing hemodialysis. Cao et al. [21] reported that depression status was associated with a higher risk of cognitive impairment compared with non-depressed individuals (OR = 1.05; 95% CI: 1.01–1.10).

Oh et al. [23] reported a significant association between depression and cognitive impairment using a pooled point estimate (0.22; 95% CI: 0.16–0.28). Cao et al. [21] further reported that psychological status was strongly associated with cognitive impairment (OR = 6.01; 95% CI: 1.91–17.32), whereas hope level was significantly associated (OR = 0.01; 95% CI: 0.0–0.16).

Cheng et al. [14] also reported a strong association between depression and cognitive frailty (OR = 3.26; 95% CI: 1.91–5.57). Wei et al. [15] found that depression assessed using the Hospital Anxiety and Depression Scale (HADS) was associated with elevated risk (OR = 4.66; 95% CI: 2.98–7.27). Higher scores on the Geriatric Depression Scale (GDS-15) were associated with increased risk (OR = 1.27; 95% CI: 0.95–1.70), although the confidence interval included unity. Depression measured using the Self-Rating Depression Scale (SDS) was not statistically significant (OR = 1.50; 95% CI: 0.54–4.22), and one pooled estimate showed considerable uncertainty (OR = 0.59; 95% CI: 0.01–26.28).

Overall, depressive symptoms and broader psychological distress appear consistently associated with increased risk of cognitive impairment and cognitive frailty among patients undergoing hemodialysis, although effect sizes varied across assessment tools and analytical methods (Table 2).

### 3.6. Cardiovascular and hemodynamic factors

#### 3.6.1. Disease

Five meta-analyses assessed disease-related factors associated with cognitive impairment and cognitive frailty in patients undergoing hemodialysis. For cognitive impairment, Cao et al. [21] reported that hypertension was associated with an increased risk of cognitive impairment (OR = 4.36; 95% CI: 1.56–12.15), while Liu et al. [22] reported a smaller association (RR = 1.06; 95% CI: 0.97–1.15). Oh et al. [23] reported no clear association, with a pooled point estimate of 0.09 (95% CI: −0.07 to 0.24). For diabetes, Cao et al. [21] reported a significant association (OR = 2.12; 95% CI: 1.52–2.96), and Liu et al. [22] also reported a positive association (RR = 1.33; 95% CI: 1.04–1.71). Oh et al. [23] reported no statistically significant relationship (point estimate = 0.03; 95% CI: −1.13 to 1.18). Cardiovascular conditions were also assessed. Cao et al. [21] reported heart disease as a risk factor (OR = 1.25; 95% CI: 0.96–1.62), whereas Liu et al. [22] found no significant association for coronary heart disease (RR= 0.86; 95% CI: 0.61–1.21). Oh et al. [23] reported a positive point estimate for overall cardiovascular disease (0.14; 95% CI: −0.11-0.26). Associations were more consistent for cerebrovascular conditions. Cao et al. [21] reported a significant association between cerebrovascular accident and cognitive impairment (OR = 1.9; 95% CI: 1.39–2.60). Liu et al. [22] reported a significant association for stroke (RR = 1.66; 95% CI: 1.08–2.55).

For cognitive frailty, Cheng et al. [14] and Wei et al. [15] reported that stroke history was associated with increased risk (OR = 2.94; 95% CI: 1.72–5.03; and OR = 2.92; 95% CI: 1.53–5.57, respectively). Overall, diabetes and cerebrovascular conditions, particularly stroke history, were consistently associated with cognitive-related outcomes among patients undergoing HD. However, the strength and consistency of associations for hypertension and cardiovascular diseases varied across meta-analyses and outcome definitions (Table 2).

#### 3.6.2. Hemodynamic indicators

One meta-analysis evaluated hemodynamic indicators associated with cognitive impairment in patients undergoing hemodialysis. Cao et al. [21] reported that higher systolic blood pressure variability (SBPV) was significantly associated with increased risk of cognitive impairment (OR = 1.17; 95% CI: 1.05–1.30), whereas mean systolic blood pressure was not significantly associated (OR = 0.94; 95% CI: 0.87–1.01). The study also reported increased arterial stiffness as a significant risk factor (OR = 1.38; 95% CI: 1.23–1.56). Overall, indicators reflecting blood pressure variability and vascular stiffness were associated with a higher risk of cognitive impairment in patients undergoing hemodialysis, whereas mean systolic blood pressure alone showed no clear association (Table 2).

#### 3.6.3. Comorbidity burden

Three included meta-analyses investigated the relationship between comorbidity burden and cognitive-related outcomes (cognitive impairment and cognitive frailty) in hemodialysis populations. For cognitive impairment, Cao et al. [21] found that the presence of multiple comorbid conditions was significantly associated with a higher risk of cognitive impairment (OR = 3.80; 95% CI: 2.35–6.15).

For cognitive frailty, Wei et al. [15] reported that overall comorbidities (OR = 2.18; 95% CI: 1.22–3.90) and dialysis-related complications (OR = 2.13; 95% CI: 1.34–3.40) were both significantly associated with an increased risk. In addition, a higher Charlson Comorbidity Index (CCI) score was linked to increased likelihood of cognitive impairment (OR = 2.67; 95% CI: 1.66–4.32). Cheng et al. [14] also demonstrated that the presence of complications was significantly associated with cognitive frailty (OR = 1.93; 95% CI: 1.47–2.54).

Overall, greater comorbidity burden, including multiple chronic conditions, dialysis-related complications, and higher CCI scores, was consistently associated with cognitive-related outcomes among patients undergoing hemodialysis, although the specific associations should be interpreted according to the outcome definitions evaluated in each meta-analysis (Table 2).

### 3.7. Kidney disease and dialysis-related factors

#### 3.7.1. Dialysis parameters

Four included meta-analyses examined dialysis-related parameters associated with cognitive impairment and cognitive frailty in hemodialysis populations. For cognitive impairment, Cao et al. [21] reported that longer hemodialysis duration was significantly associated with increased risk (OR = 1; 95% CI: 0.98–1.03). Oh et al. [23] reported a pooled point estimate close to null (0; 95% CI: −0.06 to 0.06). Liu et al. [22] observed a significant association between longer time on dialysis and cognitive impairment (SMD= 0.42; 95% CI: −0.06-0.89). Dialysis adequacy and frequency were evaluated by Cao et al. [21], who reported that insufficient dialysis adequacy was not significantly associated with cognitive impairment (OR = 0.68; 95% CI: 0.46–1.01), and dialysis frequency also showed no clear association (OR = 0.65; 95% CI: 0.20–2.11).

For cognitive frailty, Cheng et al. [14] found a non-significant association (OR = 1.53; 95% CI: 0.96–2.41). Wei et al. [15] found that dialysis age was significantly associated with increased risk (OR = 2.30; 95% CI: 1.49–3.53). Hemodynamic and treatment-related parameters were also examined. Post-dialysis diastolic pressure (OR = 2.23; 95% CI: 1.25–3.98), interdialytic hypotension (OR = 2.42; 95% CI: 1.53–4.21), and low-flux hemodialysis (OR = 4.16; 95% CI: 1.97–8.79) were significantly associated with higher risk. In contrast, nocturnal hemodialysis appeared to be associated with lower risk (OR = 0.41; 95% CI: 0.17–0.87). Excessive body weight changes before dialysis were also significantly associated (OR = 0.05; 95% CI: 0.01–0.42), while dry weight was not significantly related (OR = 0.96; 95% CI: 0.93–1.01).

Overall, dialysis-related parameters showed variable associations with cognitive-related outcomes among patients undergoing HD. Longer dialysis duration and certain dialysis-related hemodynamic and modality factors may be associated with cognitive impairment or cognitive frailty; however, findings varied across outcome definitions and individual meta-analyses (Table 2).

#### 3.7.2. Renal function markers

Four included meta-analyses examined renal function markers associated with cognitive impairment and cognitive frailty in hemodialysis populations. For cognitive impairment, Cao et al. [21] reported no significant association between creatinine and cognitive impairment (OR = 1.00; 95% CI: 0.92–1.10). Oh et al. [23] likewise reported no clear association based on a pooled point estimate of 0.09 (95% CI: −0.03 to 0.20). For blood urea nitrogen (BUN), the same study also found no significant association (point estimate= −0.02; 95% CI: −0.11 to 0.07). In contrast, Cao et al. [21] identified BUN was associated with a higher risk (OR = 1.01; 95% CI: 0.97–1.05) and cystatin C as a significant risk factor for cognitive impairment (OR = 4.08; 95% CI: 1.78–9.33). Residual urine volume was associated with a lower risk (OR = 0.236; 95% CI: 0.07–0.82), whereas chronic glomerulonephritis showed no significant association with cognitive impairment (OR = 0.85; 95% CI: 0.62–1.17).

For cognitive frailty, Cheng et al. [14] and Wei et al. [15] found no significant relationship between serum creatinine levels and cognitive frailty (OR = 1.00; 95% CI: 0.99–1.00).

Overall, traditional renal function markers such as creatinine and BUN showed no consistent associations with cognitive-related outcomes among patients undergoing hemodialysis, whereas cystatin C and reduced residual renal function appeared to be more closely associated with cognitive impairment (Table 2).

### 3.8. Metabolic, nutritional, and inflammatory markers

Five included meta-analyses assessed metabolic, nutritional, and inflammatory markers in relation to cognitive impairment and cognitive frailty in patients undergoing hemodialysis. Hemoglobin levels were examined in several studies. For cognitive impairment, Cao et al. [21] reported that lower hemoglobin levels were significantly associated with increased risk (OR = 1.11; 95% CI: 1.05–1.17). Oh et al. [23] also reported a significant pooled point estimate for hemoglobin (0.10; 95% CI: 0.04–0.16). Serum albumin was likewise commonly evaluated. Cao et al. [21] reported a protective association for higher serum albumin levels (OR = 0.84; 95% CI: 0.72–0.99). Liu et al. [22] reported a non-significant negative association for albumin (SMD = −0.20; 95% CI: −0.42 to 0.03). Elevated CRP (OR = 1.76; 95% CI: 0.55–5.66) and homocysteine (OR = 1.88; 95% CI: 0.69–5.07) were not consistently significant. Serum uric acid was associated with increased risk (OR = 3.01; 95% CI: 0.65–14.02), although with wide confidence intervals. Other markers, including 25(OH)D, PTH, sodium, potassium, phosphorus, blood urea nitrogen, erythrocyte count, and fibroblast growth factor-23, generally did not demonstrate consistent statistically significant associations.

For cognitive frailty, Cheng et al. [14] and Wei et al. [15] found no significant association for hemoglobin levels (OR = 0.67; 95% CI: 0.36–1.26; and OR = 1.00; 95% CI: 0.38–2.64, respectively). Wei et al. [15] reported a protective association for higher serum albumin levels (OR = 0.80; 95% CI: 0.66–0.90), whereas Cheng et al. [14] found no statistically significant relationship (OR = 0.72; 95% CI: 0.41–1.27). Malnutrition was consistently associated with higher risk across the studies by Cheng et al. [14] (OR = 2.91; 95% CI: 1.94–4.35) and Wei et al. [15] (OR = 2.78; 95% CI: 1.83–4.22). Triglycerides were also significantly associated with increased risk (OR = 4.03; 95% CI: 1.87–8.70).

Overall, metabolic, nutritional, and inflammatory markers showed different associations with cognitive-related outcomes among patients undergoing hemodialysis. Nutritional indicators, particularly serum albumin and malnutrition, appeared to be associated with cognitive-related outcomes, although the specific associations differed between cognitive impairment and cognitive frailty (Table 2).

### 3.9. Neurological injury biomarkers

One included meta-analysis examined neurological injury-related biomarkers associated with cognitive impairment in hemodialysis populations. Cao et al. [21] reported that elevated plasma T-tau levels were significantly associated with increased risk of cognitive impairment (OR = 1.52; 95% CI: 1.17–1.98). Neuron-specific enolase was also positively associated with cognitive impairment (OR = 12.33; 95% CI: 0.60–251.39), although the confidence interval was wide. Elevated S100B levels were associated with a higher risk (OR = 11.30; 95% CI: 1.02–124.19). Intracranial artery calcification was also associated with cognitive impairment (OR = 2.65; 95% CI: 0.49–16.04), although the estimate showed considerable uncertainty. These findings are based on limited data and require confirmation in larger studies (Table 2).

### 3.10. Body composition and fluid status

One included meta-analysis examined body composition and fluid status parameters associated with cognitive impairment in hemodialysis populations. Cao et al. [21] reported that lower lean tissue mass was significantly associated with an increased risk of cognitive impairment (OR = 5.28; 95% CI: 1.03–27.15). Total body water was also positively associated with cognitive impairment (OR = 1.68; 95% CI: 0.32–8.75), although the confidence interval was wide and crossed unity. In addition, elevated extracellular water showed a positive association (OR = 4.42; 95% CI: 0.75–26.18), but with considerable statistical uncertainty (Table 2). Overall, alterations in body composition-particularly reduced lean tissue mass-and fluid imbalance may be related to cognitive impairment in patients undergoing hemodialysis; however, the evidence is limited and warrants further investigation in larger studies (Table 2).

### 3.11. Hematologic inflammatory ratios

One included meta-analysis examined hematologic inflammatory ratios associated with cognitive impairment in hemodialysis populations. Cao et al. [21] reported that higher neutrophil percentage was significantly associated with increased risk of cognitive impairment (OR = 1.156; 95% CI: 1.06–1.27). Similarly, elevated platelet-to-lymphocyte ratio (PLR) was strongly associated with higher risk (OR = 2.73; 95% CI: 1.90–3.91). In addition, increased neutrophil-to-lymphocyte ratio (NLR) was significantly related to cognitive impairment (OR = 1.89; 95% CI: 1.31–2.72). Overall, elevated systemic inflammatory markers derived from routine hematologic indices appear to be associated with a higher likelihood of cognitive impairment in patients undergoing hemodialysis, suggesting a potential role of inflammation in the underlying pathophysiology (Table 2).

### 3.12. Assessment of overlap among included meta-analyses

The pairwise overlap matrix generated using the CCA calculator indicated that overlap among the included meta-analyses was generally low. Most pairwise overlap values ranged from 0.0% to 4.4%, suggesting no or only slight overlap between reviews. Specifically, slight overlap was observed between Liu 2023 and Cao 2022 (4.4%), Oh 2019 and Cao 2022 (2.7%), Oh 2019 and Cheng 2024 (1.9%), and Oh 2019 and Liu 2023 (2.1%). The highest overlap was found between Wei 2025 and Cheng 2024 (11.1%), indicating a high degree of overlap. All other pairwise comparisons showed no overlap (0.0%). Overall, these findings suggest limited redundancy of primary studies across most included reviews, although the relatively high overlap between Wei 2025 and Cheng 2024 should be taken into account when interpreting the pooled evidence (Figure 5; Table S2).

### 3.13. Evidence heatmap and radar chart

Figure 3 presents the evidence heatmap summarizing the evidence profiles of factors associated with cognitive impairment and cognitive frailty in hemodialysis patients. Most factors were positively associated with cognitive impairment and cognitive frailty, whereas educational level showed a protective association. Age-related indicators (every year increase, older age, and age) demonstrated the strongest statistical significance and higher evidence levels. Hemoglobin level also showed strong statistical significance. Consistency across studies was generally limited, with only complication, dialysis age, and malnutrition showing better consistency.

**Figure 3.**
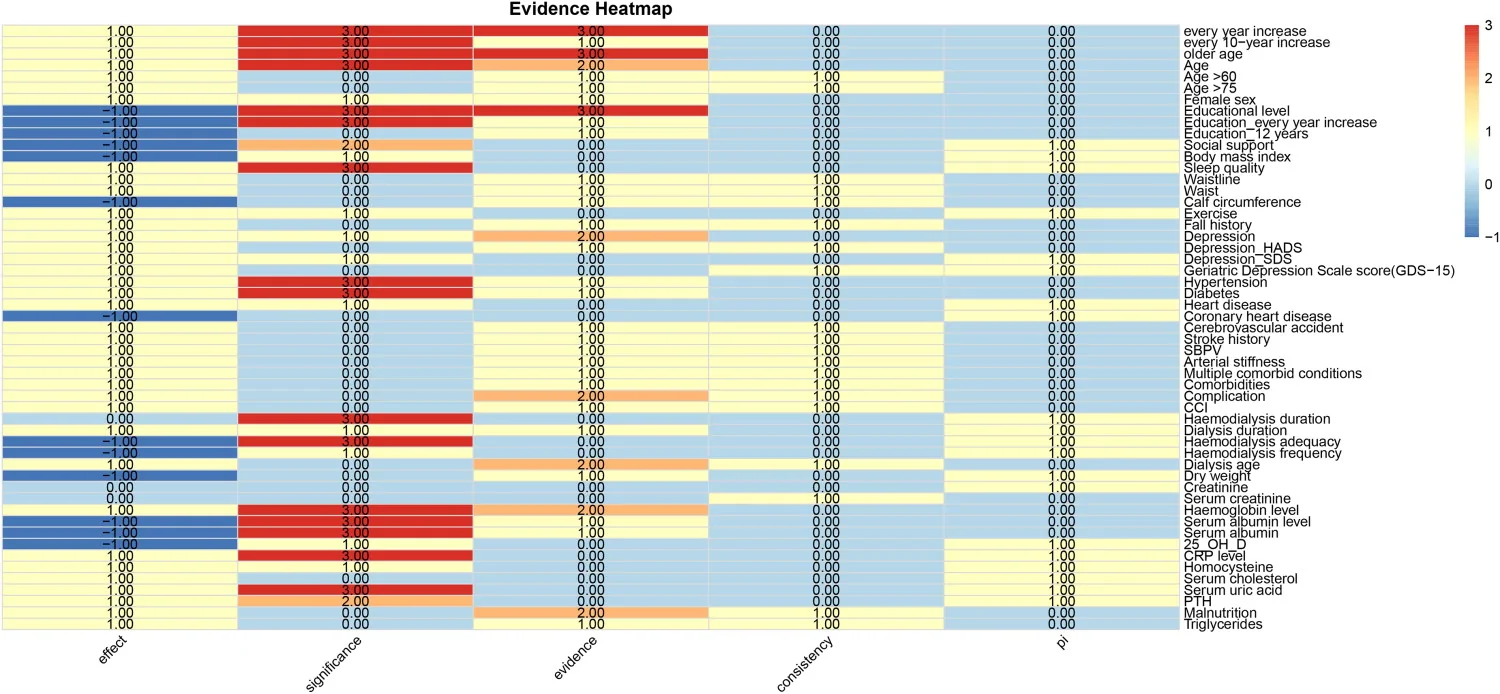
Evidence heatmap summarizing the strength and consistency of evidence for risk factors associated with cognitive impairment. Colors correspond to evidence scores, ranging from negative or inconsistent evidence (blue) to stronger evidence (red).

Figure 4 presents the radar plot of selected factors. Age-related variables showed the highest statistical significance and evidence levels, whereas complication, dialysis age, and malnutrition showed relatively better consistency but weaker statistical significance.

**Figure 4.**
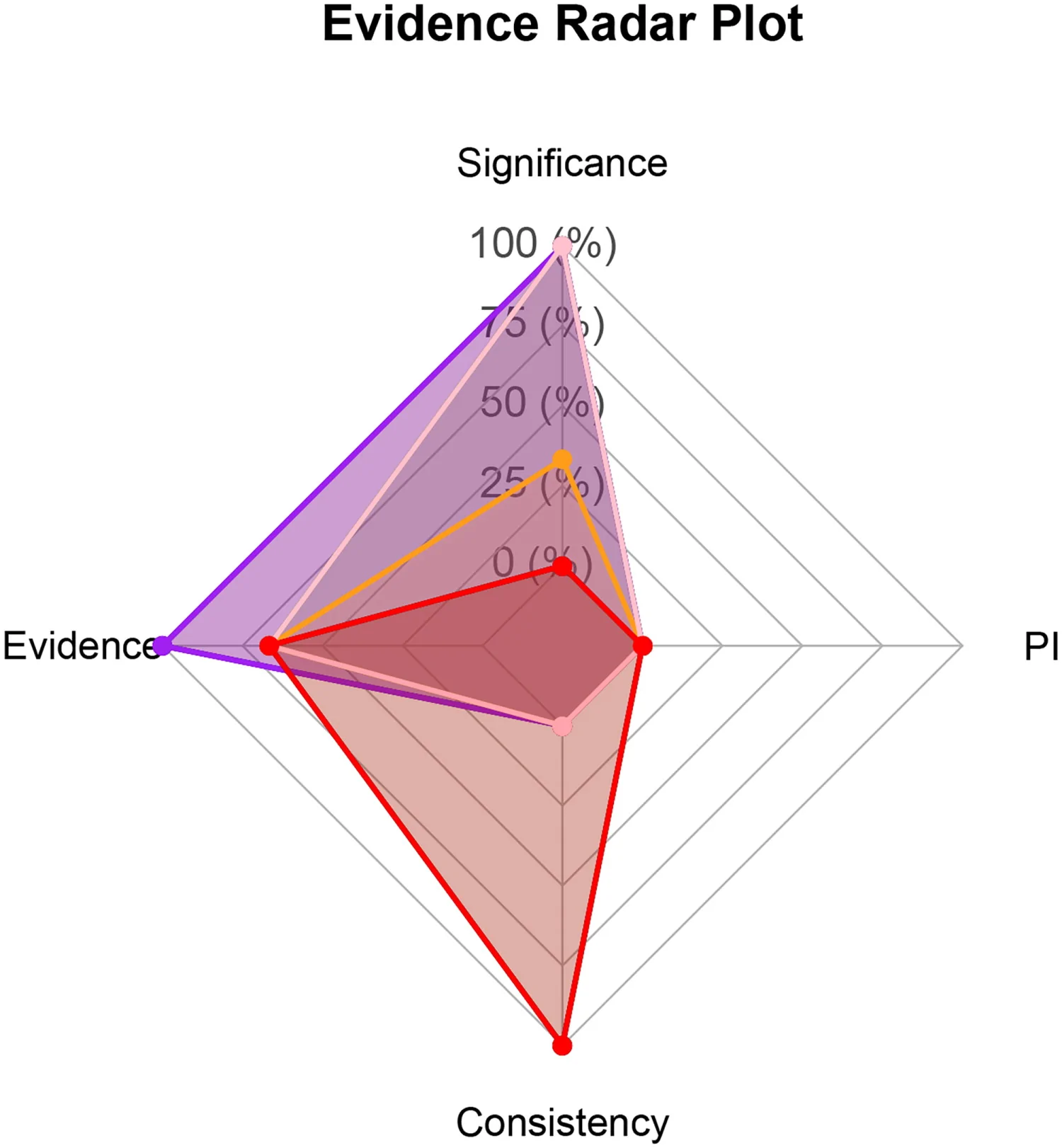
Radar plot summarizing evidence profiles of risk factors associated with cognitive impairment. Each axis represents an evidence domain, and different colors indicate individual risk factors or evidence profiles. Higher values indicate stronger evidence across domains.

**Figure 5.**
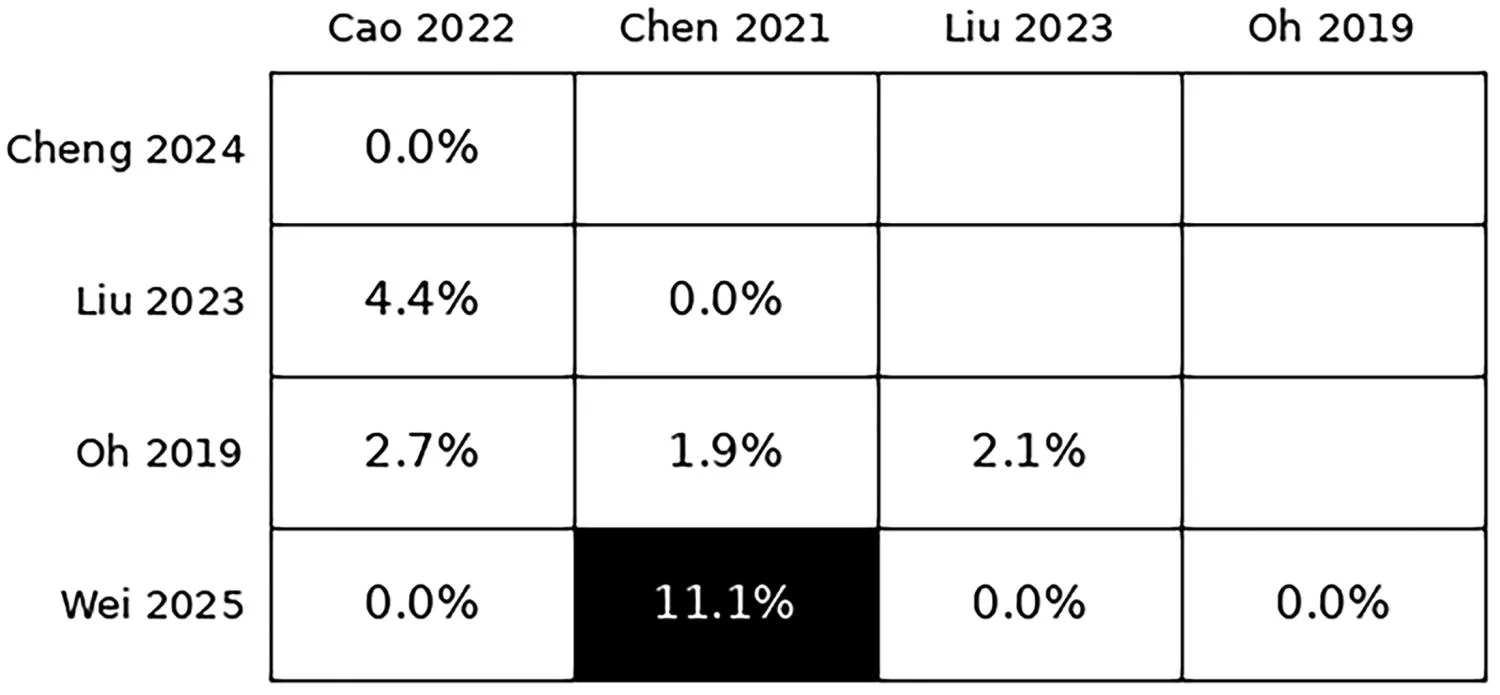
Corrected Covered Area (CCA) matrix showing the percentage overlap of primary studies among included meta-analyses.

### 3.14. Heterogeneity

Reanalysis showed that 42.1% of the included studies demonstrated significant heterogeneity, indicated by I^2^ values exceeding 50% or Cochran’s Q-test *P* values below 0.10. Such variability may be attributable to differences in study setting, geographic region, ethnicity, participant characteristics (e.g. age and sex), study quality, design, sample size, follow-up duration, and adjustment for confounding factors. Furthermore, 27.8% of the studies did not report heterogeneity assessments (Table 2).

### 3.15. Assessment of risk of bias

Egger’s test was employed to evaluate publication bias in 7.1% of the included studies. No significant publication bias was identified; however, 27.8% of the included studies did not report bias assessment findings (Table 2).

### 3.16. AMSTAR-2 evaluation

The methodological quality of the five included systematic reviews was assessed using AMSTAR-2. All reviews were rated as low confidence. Most reviews demonstrated adequate methodological practices, including clearly defined research questions and eligibility criteria, comprehensive literature searches, duplicate study selection and data extraction, appropriate risk of bias assessment, appropriate statistical synthesis, and evaluation of heterogeneity and publication bias. Nevertheless, important methodological weaknesses were identified. None of the included reviews provided a list of excluded full-text studies with reasons for exclusion (Q7) or reported the funding sources of individual included studies (Q10). Furthermore, although review methods were generally described, protocol-related requirements were incompletely reported, particularly predefined meta-analysis plans and plans for exploring heterogeneity (Q2). These limitations reduced the overall confidence in the evidence synthesized by the included reviews (Table S3).

### 3.17. Grade rating

According to the GRADE framework, only two associations-place of residence (hospitals) and history of hip fracture-were rated as moderate-quality evidence. All other identified factors were graded as low or very low certainty (Table S4). The low certainty largely reflects the observational design of the primary studies included in the meta-analyses. Accordingly, although several associations reached statistical significance, the evidence should be interpreted cautiously.

## 4. Discussion

This study systematically synthesized the existing evidence on factors associated with cognitive impairment in patients receiving maintenance hemodialysis. In this umbrella review, cognitive impairment was considered the primary outcome, whereas cognitive frailty identified in some included meta-analyses was treated as a separate cognitive-related outcome rather than an interchangeable term for cognitive impairment. Cognitive frailty represents a composite construct involving cognitive impairment and physical frailty and was therefore interpreted separately according to the original definitions used in the included reviews. Cognitive impairment in patients receiving maintenance hemodialysis is associated with multiple factors, including demographic characteristics (advanced age, female sex, and lower educational attainment), vascular comorbidities (history of stroke, hypertension, and diabetes), dialysis-related factors (longer dialysis duration and biochemical abnormalities), nutritional and metabolic indicators (low hemoglobin, reduced albumin, and altered creatinine and lipid levels), and psychosocial factors such as depression and sedentary behavior. For cognitive frailty, additional factors related to physical function and frailty characteristics, including falls, impaired mobility, and functional limitations, were identified in the corresponding meta-analyses but were not considered general risk factors for cognitive impairment. Rather than merely confirming previously reported associations, this umbrella review integrated evidence from multiple meta-analyses and evaluated the consistency, magnitude, and credibility of these associations using standardized evidence classification approaches. Several factors showed consistent associations across pooled analyses, reflecting interactions among vascular injury, metabolic disturbances, and neurodegeneration. In particular, advanced age, lower educational attainment, depression, stroke history, diabetes, and selected nutritional or metabolic indicators appeared to represent more reproducible associated factors, whereas several dialysis-related and biochemical factors were supported by less consistent evidence. Associations identified for cognitive frailty, including physical performance-related factors, should be interpreted within the context of this distinct construct rather than extrapolated to cognitive impairment alone. Cognitive impairment is highly prevalent among patients undergoing hemodialysis, with the reported rates often exceeding 40% [1,2]. Impairments in executive and memory functions have been shown to independently predict mortality [2,3]. Cognitive impairment and cognitive frailty are both clinically meaningful conditions in dialysis populations, but they represent different constructs and may have distinct prognostic implications. Cognitive impairment in hemodialysis patients is a clinically meaningful complication with prognostic implications that warrants systematic risk stratification. Importantly, the findings of this umbrella review should be interpreted as evidence for identifying higher-priority factors for risk assessment rather than establishing causal relationships. These findings help clarify which routinely reported clinical factors may be most informative for risk identification, rather than treating all reported associations as equally reliable.

Age was the most consistent associated factor across studies, with increasing age associated with cognitive decline in dialysis populations [24,25]. Advanced age was independently associated with poorer cognitive performance [7]. Longitudinal data also showed progressive declines in MMSE scores over 5 years among elderly HD patients, with age independently predicting mortality [3]. Educational attainment, a proxy for cognitive reserve, showed a protective effect in our analysis and was associated with lower cognitive frailty and executive dysfunction [14,26]. Because cognitive frailty was evaluated separately from cognitive impairment, the protective association of education with cognitive frailty should be interpreted as evidence related to this specific construct rather than as direct evidence for cognitive impairment alone. Xia et al. [27] reported that cognitive reserve scores showed strong ability to identify cognitive frailty. The consistent association of age and education suggests that cognitive impairment in HD is not explained solely by dialysis exposure, but also by accumulated vulnerability and reduced cognitive reserve. However, these findings should not be interpreted as direct causal effects, as age-related biological vulnerability and educational attainment may interact with multiple unmeasured socioeconomic, vascular, and health-related factors. These findings suggest that neurodegeneration in HD patients reflects cumulative vascular and metabolic insults acting on underlying cognitive reserve.

Vascular and metabolic factors were major risk domains. Prior stroke and cerebrovascular disease were consistently associated with cognitive impairment [7,8]. Executive dysfunction has been reported in more than half of patients receiving HD [11,28]. Neuroimaging studies show that gray matter atrophy and hippocampal microstructural abnormalities correlate with declines in MoCA and MMSE scores [4,5]. Higher hemoglobin levels were associated with a protective effect, whereas elevated parathyroid hormone and uremic toxins were associated with increased risk [1,4]. These findings are biologically plausible, as anemia-related cerebral hypoxia and toxin-induced endothelial dysfunction may contribute to neuronal injury [9,10]. Emerging biomarkers, including HIF-1α and protein-bound toxins such as indoxyl sulfate and homocysteine, indicate the involvement of hypoxic and inflammatory pathways [29,30]. However, the strength of evidence differed across these factors, indicating that biologically plausible mechanisms do not necessarily translate into equally robust epidemiological associations. Furthermore, most associations were supported by low or very low certainty evidence, and residual confounding may partly explain the observed relationships. These findings suggest that cognitive impairment in HD patients reflects vascular pathology, uremic neurotoxicity, and dialysis-related hemodynamic stress.

Psychosocial and lifestyle factors also showed associations. Depression was linked to increased cognitive vulnerability [1,14,31]. Depressive symptoms and executive dysfunction may share inflammatory and neuroendocrine mechanisms. Sedentary behavior was also associated with poorer performance on cognitive tests [12]. Physical function-related factors, including gait speed, grip strength, falls, and activities of daily living, were mainly reported in relation to cognitive frailty or specific functional outcomes and should not be interpreted as universal risk factors for cognitive impairment. Frailty and cognitive frailty in HD populations have been associated with increased risks of mortality and hospitalization [2,26,32]. Nutritional indicators, including albumin, cholesterol, and creatinine levels, were associated with cognitive function [13,33]. Lower cholesterol and creatinine levels, often reflecting malnutrition or sarcopenia, were linked to poorer cognitive performance. These potentially modifiable factors may be particularly relevant for clinical screening because they connect cognitive impairment with depression, frailty, physical inactivity, and nutritional decline, which are common but often underassessed in dialysis care. These findings indicate that cognitive decline in HD patients involves biological, behavioral, and social factors. Nevertheless, whether modification of these factors can prevent or reverse cognitive impairment remains uncertain because available evidence is primarily observational. Future intervention studies are required to determine whether these potentially modifiable factors represent true therapeutic targets.

All studies included in this umbrella review and meta-analysis were observational, including cross-sectional and cohort designs, which limits causal inference and leaves the possibility of residual confounding. Heterogeneity in cognitive assessment tools (MoCA, MMSE, ACE-III, 3MS) and differences in diagnostic thresholds may have introduced variability in effect estimates. Publication bias and selective reporting cannot be fully excluded. In addition, most studies were conducted in single countries or specific regions, which may limit generalizability across different populations and healthcare settings. Randomized controlled trials evaluating targeted interventions remain limited (6, 8). Therefore, although this study identifies several factors with relatively consistent evidence, these findings should be interpreted as evidence for risk stratification rather than proof of causality. In addition, the overall certainty of evidence for many associations was limited, indicating that statistically significant associations should be interpreted cautiously. Reverse causation may also exist, particularly for factors such as nutritional status, physical activity, and depressive symptoms, where cognitive impairment itself may influence these conditions. Future studies should prioritize prospective designs, standardized cognitive assessment, and intervention studies targeting modifiable risk factors.

## 5. Conclusion

Cognitive impairment is common among patients receiving maintenance hemodialysis and is associated with multiple factors. In addition, cognitive frailty represents a related but distinct condition involving both cognitive impairment and physical frailty, and findings from cognitive frailty studies should be interpreted separately rather than combined directly with cognitive impairment evidence. This study further clarified that these factors should not be interpreted as equivalent in evidential strength, because some associations were more consistent and clinically informative than others. Advanced age, lower educational attainment, and female sex were demographic associated factors, while cerebrovascular disease, diabetes, and hypertension were important vascular factors. Dialysis-related and metabolic disturbances, including anemia, mineral imbalance, and uremic toxin accumulation, were also associated with increased risk. Depression, sedentary behavior, and malnutrition were identified as potentially modifiable factors. Factors related to physical function and frailty, including gait performance, falls, and functional dependence, were interpreted within the context of cognitive frailty rather than as general risk factors for cognitive impairment. By integrating these reported associations across demographic, vascular, dialysis-related, metabolic, and psychosocial domains, our findings provide a more structured basis for risk stratification in hemodialysis populations. Compared with individual meta-analyses focusing on isolated factors, this umbrella review further evaluated the consistency and credibility of available evidence, demonstrating that statistical significance alone does not necessarily indicate reliable evidence. However, because the included studies were observational, causal relationships cannot be definitively established. Moreover, the generally low or very low certainty of evidence highlights the need for cautious interpretation, and residual confounding and reverse causation should be considered. Future prospective studies are needed to determine whether modifying these higher-priority associated factors can reduce the burden of cognitive impairment in this population.

## Supplementary Material

Supplemental Material

Supplemental Material

Supplemental Material

Supplemental Material

## Data Availability

All data relevant to this study are included in the article and its supplementary materials. And all the supplementary was uploaded in OSF data repository, the link is https://osf.io/xvkw9/overview?view_only=fcf3acfc3331457a806722e1bad619b4.

## References

[1] Tian X, Xia X, Yu H, et al. Cognitive dysfunction and its risk factors in patients undergoing maintenance hemodialysis. Neuropsychiatr Dis Treat. 2022;18:2759–2769. doi: 10.2147/NDT.S380518. PMC9704003 36452115

[2] Guo Y, Tian R, Ye P, et al. Cognitive domain impairment and all-cause mortality in older patients undergoing hemodialysis. Front Endocrinol (Lausanne)). 2022;13:828162 doi: 10.3389/fendo.2022.828162. PMC8995766 35418951

[3] Miyazaki S, Kitamura M, Hayashida M, et al. Survival and cognitive deterioration in elderly patients undergoing hemodialysis. Geriatr Gerontol Int. 2023;23(2):111–116. doi: 10.1111/ggi.14531. 36608644

[4] Wang H, Huang L, Wu G, et al. Regional cerebral gray matter atrophy is associated with cognitive impairment in hemodialysis patients: a cross-sectional and longitudinal voxel-based morphological MRI study. Brain Imaging Behav. 2022;16(3):1284–1293. doi: 10.1007/s11682-021-00602-9. 34993881

[5] Zheng J, Jiao Z, Dai J, et al. Abnormal cerebral micro-structures in end-stage renal disease patients related to mild cognitive impairment. Eur J Radiol. 2022;157:110597 doi: 10.1016/j.ejrad.2022.110597. 36379097

[6] Kanbay M, Mizrak B, Copur S, et al. Cognitive impairment in patients on dialysis: can we prevent it? J Nephrol. 2024;37(7):1813–1820. doi: 10.1007/s40620-024-02093-5. 39305447

[7] Shoji T, Fujii H, Mori K, et al. Associations of cardiovascular disease and blood pressure with cognition in hemodialysis patients: the Osaka Dialysis Complication Study. Nephrol Dial Transplant. 2022;37(9):1758–1767. doi: 10.1093/ndt/gfab247. 34459921

[8] Tsuruya K, Yoshida H. Cognitive impairment and brain atrophy in patients with chronic kidney disease. J Clin Med. 2024;13(5):1401. doi: 10.3390/jcm13051401. PMC10931800 38592226

[9] Bolignano D, Simeoni M, Hafez G, et al. Cognitive impairment in CKD patients: a guidance document by the CONNECT network. Clin Kidney J. 2025;18(2):sfae294 doi: 10.1093/ckj/sfae294. PMC11997768 40235626

[10] Cozza M, Boccardi V. Cognitive impairment in chronic kidney disease: mechanisms, clinical manifestations, and management strategies. Metab Brain Dis. 2025;40(8):312 doi: 10.1007/s11011-025-01735-2. 41201682

[11] Belluardo G, Galeano D, Sessa C, et al. Executive dysfunction in patients undergoing chronic haemodialysis treatment: a possible symptom of vascular dementia. G Ital Nefrol. 2024;41(2):72–87. 38695231

[12] Golenia A, Olejnik P, Maciejewska O, et al. Sedentary lifestyle is a modifiable risk factor for cognitive impairment in patients on dialysis and after kidney transplantation. J Clin Med. 2024;13(20):6083. doi: 10.3390/jcm13206083. PMC11508775 39458033

[13] Li S, Huang L, Wang W, et al. Prevalence and influence factors of cognitive frailty in the older adult patients undergoing maintenance haemodialysis: a multi-centre cross-sectional study. J Clin Nurs. 2025;34(9):3693–3703. doi: 10.1111/jocn.17527. 39497623

[14] Cheng M, Liu Q, Gan H, et al. Prevalence and risk factors of cognitive frailty in patients undergoing maintenance hemodialysis: a systematic review and meta-analysis. Semin Dial. 2024;37(5):363–372. doi: 10.1111/sdi.13220. 39120010

[15] Wei X, Peng J, Chang R, et al. Prevalence and influencing factors of cognitive frailty in Chinese maintenance hemodialysis patients: a systematic review and meta-analysis. BMC Nephrol. 2025;26(1):365. doi: 10.1186/s12882-025-04288-9. PMC12235981 40629315

[16] Page MJ, McKenzie JE, Bossuyt PM, et al. The PRISMA 2020 statement: an updated guideline for reporting systematic reviews. BMJ. 2021;372:n71. doi: 10.1136/bmj.n71. PMC8005924 33782057

[17] Santos WMD, Secoli SR, Püschel VAA. The Joanna Briggs Institute approach for systematic reviews. Rev Lat Am Enfermagem. 2018;26:e3074. doi: 10.1590/1518-8345.2885.3074. PMC6248737 30462787

[18] Cumpston M, Li T, Page MJ, et al. Updated guidance for trusted systematic reviews: a new edition of the Cochrane Handbook for Systematic Reviews of Interventions. Cochrane Database Syst Rev. 2019;10(10):Ed000142. doi: 10.1002/14651858.ED000142. PMC10284251 31643080

[19] Shea BJ, Reeves BC, Wells G, et al. AMSTAR 2: a critical appraisal tool for systematic reviews that include randomised or non-randomised studies of healthcare interventions, or both. BMJ. 2017;358:j4008. doi: 10.1136/bmj.j4008. PMC5833365 28935701

[20] Aguayo-Albasini JL, Flores-Pastor B, Soria-Aledo V. GRADE system: classification of quality of evidence and strength of recommendation. Cir Esp. 2014;92(2):82–88. doi: 10.1016/j.ciresp.2013.08.002. 24361098

[21] Cao T, An R, Wang Y, et al. Risk factors and prevalence of cognitive impairment in maintenance haemodialysis patients: a systematic review and meta-analysis of observational studies. J Adv Nurs. 2023;79(10):3691–3706. doi: 10.1111/jan.15746. 37357980

[22] Liu J, Chen K, Chen J, et al. Incidence and risk factors of cognitive dysfunction in hemodialysis patients: a systematic review and meta-analysis. Semin Dial. 2023;36(5):358–365. doi: 10.1111/sdi.13171. 37592399

[23] Oh H, Mo J, Seo W. Correlates of cognitive impairment in patients with chronic kidney failure on haemodialysis: systematic review and meta-analysis. J Adv Nurs. 2019;75(5):962–978. doi: 10.1111/jan.13907. 30407656

[24] Chen D, Xiao C, Xiao W, et al. Prediction model for cognitive impairment in maintenance hemodialysis patients. BMC Neurol. 2023;23(1):367. doi: 10.1186/s12883-023-03407-z. PMC10568884 37828422

[25] Chen Z, Zhou K, Tan X, et al. Development and validation of a predictive model for cognitive impairment in patients undergoing maintenance hemodialysis: A retrospective, cross-sectional study. Medicine (Baltimore)). 2026;105(3):e45594. doi: 10.1097/MD.0000000000045594. PMC12826182 41560023

[26] Cheng M, He M, Ning L, et al. Association between frailty and adverse outcomes in patients undergoing maintenance hemodialysis: a systematic review and meta-analysis. Ren Fail. 2024;46(2):2367716. doi: 10.1080/0886022X.2024.2367716. PMC11740690 39099468

[27] Xia NN, Wang HY, Liu J. The effect of cognitive reserve on the risk of cognitive frailty in older patients receiving maintenance hemodialysis: a cross-sectional study. Eur Geriatr Med. 2025;16(5):1657–1665. doi: 10.1007/s41999-025-01260-6. 40550994

[28] Olczyk P, Kusztal M, Gołębiowski T, et al. Cognitive impairment in end stage renal disease patients undergoing hemodialysis: markers and risk factors. Int J Environ Res Public Health. 2022;19(4):2389. doi: 10.3390/ijerph19042389. PMC8877881 35206577

[29] Guo L, Jia C, Luo K, et al. Elevated HIF-1α levels in maintenance hemodialysis patients: a potential link to increased cognitive impairment risk. Front Aging Neurosci. 2024;16:1455596. doi: 10.3389/fnagi.2024.1455596. PMC11663881 39717345

[30] Wang M, Hua W, Chen L, et al. Enhanced cognitive outcomes with triple-mode dialysis in kidney failure: role of protein-bound toxin clearance. Ren Fail. 2025;47(1):2590874. doi: 10.1080/0886022X.2025.2590874. PMC12649770 41290558

[31] Golenia A, Żołek N, Olejnik P, et al. Patterns of cognitive impairment in hemodialysis patients and related factors including depression and anxiety. J Clin Med. 2023;12(9):3119. doi: 10.3390/jcm12093119. PMC10179667 37176560

[32] Guo Y, Tian R, Ye P, et al. Frailty in older patients undergoing hemodialysis and its association with all-cause mortality: a prospective cohort study. Clin Interv Aging. 2022;17:265–275. doi: 10.2147/CIA.S357582. PMC8934156 35313671

[33] Song Y, Lu J, Wu Y, et al. Factors influencing cognitive impairment in patients undergoing hemodialysis: based on health ecological model. Dement Geriatr Cogn Disord. 2026;55(3):173–186. doi: 10.1159/000547516. 40763667

