# Supplementary material for: Risk factors for cognitive impairment in patients undergoing hemodialysis: an umbrella review and meta-analysis
Source: Ren Fail. 2026 Nov 3;48(1):2741985. doi: 10.1080/0886022X.2026.2741985 (PMC13757910; doi:10.1080/0886022X.2026.2741985)
Supplement: Supplementary file 1 [file IRNF_A_2741985_SM8704.docx]

| **Table S1** Searching strategy | | |
| --- | --- | --- |
| Databases | Strategy | Literature number |
| Pubmed | (((((((((((((((Renal Dialysis[MeSH Terms]) OR (Dialysis[MeSH Terms])) OR (Renal Dialysis[Title/Abstract])) OR (Dialyses, Renal[Title/Abstract])) OR (Renal Dialyses[Title/Abstract])) OR (Dialysis, Renal[Title/Abstract])) OR (Hemodialysis[Title/Abstract])) OR (Hemodialyses[Title/Abstract])) OR (Dialysis, Extracorporeal[Title/Abstract])) OR (Dialyses, Extracorporeal[Title/Abstract])) OR (Extracorporeal Dialyses[Title/Abstract])) OR (Extracorporeal Dialysis[Title/Abstract])) OR (Dialysis[Title/Abstract])) OR (Dialyses[Title/Abstract])) AND (((((((((((((((((((((((((((Cognitive Dysfunction[MeSH Terms]) OR (Cognitive Dysfunction[Title/Abstract])) OR (Cognitive Dysfunctions[Title/Abstract])) OR (Dysfunction, Cognitive[Title/Abstract])) OR (Dysfunctions, Cognitive[Title/Abstract])) OR (Cognitive Disorder[Title/Abstract])) OR (Cognitive Disorders[Title/Abstract])) OR (Disorder, Cognitive[Title/Abstract])) OR (Disorders, Cognitive[Title/Abstract])) OR (Cognitive Impairments[Title/Abstract])) OR (Cognitive Impairment[Title/Abstract])) OR (Impairment, Cognitive[Title/Abstract])) OR (Impairments, Cognitive[Title/Abstract])) OR (Mild Cognitive Impairment[Title/Abstract])) OR (Cognitive Impairment, Mild[Title/Abstract])) OR (Cognitive Impairments, Mild[Title/Abstract])) OR (Impairment, Mild Cognitive[Title/Abstract])) OR (Impairments, Mild Cognitive[Title/Abstract])) OR (Mild Cognitive Impairments[Title/Abstract])) OR (Cognitive Decline[Title/Abstract])) OR (Cognitive Declines[Title/Abstract])) OR (Decline, Cognitive[Title/Abstract])) OR (Declines, Cognitive[Title/Abstract])) OR (Mental Deterioration[Title/Abstract])) OR (Deterioration, Mental[Title/Abstract])) OR (Deteriorations, Mental[Title/Abstract])) OR (Mental Deteriorations[Title/Abstract]))) AND ((meta-analysis[Title/Abstract]) OR (Systematic review[Title/Abstract])) | 37 |
| Web of science | 1 "TS=(Renal Dialysis OR Dialyses, Renal OR Renal Dialyses OR Dialysis, Renal OR Hemodialysis OR Hemodialyses OR Dialysis, Extracorporeal OR Dialyses, Extracorporeal OR Extracorporeal Dialyses OR Extracorporeal Dialysis OR Dialysis OR Dialyses) "  2 "TS=(Cognitive Dysfunction OR Cognitive Dysfunctions OR Dysfunction, Cognitive OR Dysfunctions, Cognitive OR Cognitive Disorder OR Cognitive Disorders OR Disorder, Cognitive OR Disorders, Cognitive OR Cognitive Impairments OR Cognitive Impairment OR Impairment, Cognitive OR Impairments, Cognitive OR Mild Cognitive Impairment OR Cognitive Impairment, Mild OR Cognitive Impairments, Mild OR Impairment, Mild Cognitive OR Impairments, Mild Cognitive OR Mild Cognitive Impairments OR Cognitive Decline OR Cognitive Declines OR Decline, Cognitive OR Declines, Cognitive OR Mental Deterioration OR Deterioration, Mental OR Deteriorations, Mental OR Mental Deteriorations) "  3 "TS=(meta-analysis OR Systematic review) "  4 "#3 AND #2 AND #1 " | 71 |
| Embase | #47 #15 AND #43 AND #46  #46 #44 OR #45  #45 'systematic review':ab,kw,ti  #44 'meta analysis':ab,kw,ti  #43 #16 OR #17 OR #18 OR #19 OR #20 OR #21 OR #22 OR #23 OR #24 OR #25 OR #26 OR #27 OR #28 OR #29 OR #30 OR #31 OR #32 OR #33 OR #34 OR #35 OR #36 OR #37 OR #38 OR #39 OR #40 OR #41 OR #42  #42 'mental deteriorations':ab,kw,ti  #41 'deteriorations, mental':ab,kw,ti  #40 'deterioration, mental':ab,kw,ti  #39 'mental deterioration':ab,kw,ti  #38 'declines, cognitive':ab,kw,ti  #37 'decline, cognitive':ab,kw,ti  #36 'cognitive declines':ab,kw,ti  #35 'cognitive decline':ab,kw,ti  #34 'mild cognitive impairments':ab,kw,ti  #33 'impairments, mild cognitive':ab,kw,ti  #32 'impairment, mild cognitive':ab,kw,ti  #31 'cognitive impairments, mild':ab,kw,ti  #30 'cognitive impairment, mild':ab,kw,ti  #29 'mild cognitive impairment':ab,kw,ti  #28 'impairments, cognitive':ab,kw,ti  #27 'impairment, cognitive':ab,kw,ti  #26 'cognitive impairment':ab,kw,ti  #25 'cognitive impairments':ab,kw,ti  #24 'disorders, cognitive':ab,kw,ti  #23 'disorder, cognitive':ab,kw,ti  #22 'cognitive disorders':ab,kw,ti  #21 'cognitive disorder':ab,kw,ti  #20 'dysfunctions, cognitive':ab,kw,ti  #19 'dysfunction, cognitive':ab,kw,ti  #18 'cognitive dysfunctions':ab,kw,ti  #17 'cognitive dysfunction':ab,kw,ti  #16 'cognitive dysfunction'/exp  #15 #1 OR #2 OR #3 OR #4 OR #5 OR #6 OR #7 OR #8 OR #9 OR #10 OR #11 OR #12 OR #13 OR #14  #14 'dialyses':ab,kw,ti  #13 'dialysis':ab,kw,ti  #12 'extracorporeal dialysis':ab,kw,ti  #11 'extracorporeal dialyses':ab,kw,ti  #10 'dialyses, extracorporeal':ab,kw,ti  #9 'dialysis, extracorporeal':ab,kw,ti  #8 'hemodialyses':ab,kw,ti  #7 'hemodialysis':ab,kw,ti  #6 'dialysis, renal':ab,kw,ti  #5 'renal dialyses':ab,kw,ti  #4 'dialyses, renal':ab,kw,ti  #3 'renal dialysis':ab,kw,ti  #2 'dialysis'/exp  #1 'renal dialysis'/exp | 116 |
| Cochrane | #1 (Renal Dialysis):ti,ab,kw OR (Dialyses, Renal):ti,ab,kw OR (Renal Dialyses):ti,ab,kw OR (Dialysis, Renal):ti,ab,kw OR (Hemodialysis):ti,ab,kw 21389  #2 (Hemodialyses):ti,ab,kw OR (Dialysis, Extracorporeal):ti,ab,kw OR (Dialyses, Extracorporeal):ti,ab,kw OR (Extracorporeal Dialyses):ti,ab,kw OR (Extracorporeal Dialysis):ti,ab,kw 392  #3 (Dialysis):ti,ab,kw OR (Dialyses):ti,ab,kw 19336  #4 #1 OR #2 OR #3 24880  #5 (Cognitive Dysfunction):ti,ab,kw OR (Cognitive Dysfunctions):ti,ab,kw OR (Dysfunction, Cognitive):ti,ab,kw OR (Dysfunctions, Cognitive):ti,ab,kw OR (Cognitive Disorder):ti,ab,kw 35677  #6 (Cognitive Disorders):ti,ab,kw OR (Disorder, Cognitive):ti,ab,kw OR (Disorders, Cognitive):ti,ab,kw OR (Cognitive Impairments):ti,ab,kw OR (Cognitive Impairment):ti,ab,kw 56995  #7 (Impairment, Cognitive):ti,ab,kw OR (Impairments, Cognitive):ti,ab,kw OR (Mild Cognitive Impairment):ti,ab,kw OR (Cognitive Impairment, Mild):ti,ab,kw OR (Cognitive Impairments, Mild):ti,ab,kw 23513  #8 (Impairment, Mild Cognitive):ti,ab,kw OR (Impairments, Mild Cognitive):ti,ab,kw OR (Mild Cognitive Impairments):ti,ab,kw OR (Cognitive Decline):ti,ab,kw OR (Cognitive Declines):ti,ab,kw 14554  #9 (Decline, Cognitive):ti,ab,kw OR (Declines, Cognitive):ti,ab,kw OR (Mental Deterioration):ti,ab,kw OR (Deterioration, Mental):ti,ab,kw OR (Deteriorations, Mental):ti,ab,kw 9873  #10 (Mental Deteriorations):ti,ab,kw 24  #11 #5 OR #6 OR #7 OR #8 OR #9 OR #10 65164  #12 (meta-analysis):ti,ab,kw OR (Systematic review):ti,ab,kw 30712  #13 #4 AND #11 AND #12 18 | 18 |
