# Supplementary material for: Risk factors for cognitive impairment in patients undergoing hemodialysis: an umbrella review and meta-analysis
Source: Ren Fail. 2026 Nov 3;48(1):2741985. doi: 10.1080/0886022X.2026.2741985 (PMC13757910; doi:10.1080/0886022X.2026.2741985)
Supplement: Supplementary file 2 [file IRNF_A_2741985_SM8705.docx]

| Table S2 Citation matrix used for the assessment of overlap among primary studies included in the included meta-analyses | | | | | | |
| --- | --- | --- | --- | --- | --- | --- |
| **Primary study** | **Meta-anlysis** | | | | | **Reference Titles** |
| Study ID | Cao 2022 | Cheng 2024 | Liu 2023 | Oh 2019 | Wei 2025 |  |
| Jiang 2020 | 0 | 1 | 0 | 0 | 1 | Cognitive frailty in elderly patients with Hemodialysis and its relationship with fear of falling |
| Qin 2023 | 0 | 1 | 0 | 0 | 1 | Construction and validation of risk nomograph model for cognitive frailty in maintenance Hemodialysis patients |
| Sun 2023 | 0 | 1 | 0 | 0 | 1 | Status and Influencing Factors of Cognitive Frailty in Hemodialysis Patients |
| Chen 2021W | 0 | 0 | 0 | 0 | 1 | Analysis on current status and influencing factors of cognitive frailty in maintenance Hemodialysis patients |
| Chen G 2022 | 0 | 0 | 0 | 0 | 1 | Comparison of the preva lence and associated factors of cognitive frailty between elderly and middle young patients receiving maintenance Hemodialysis |
| Li 2023W | 0 | 0 | 0 | 0 | 1 | Risk of cognitive frailty in elderly maintenance Hemodialysis patients  establishment of predictive models |
| Liu 2023Wa | 0 | 0 | 0 | 0 | 1 | Construction of Risk Prediction Model for Cognitive Frailty in Elderly of Maintenance Hemodialysis |
| Liu 2023Wb | 0 | 0 | 0 | 0 | 1 | Investigation and analysis of the current situation and influencing factors of cognitive frailty in maintenance Hemodialysis patients in nanjing |
| Wang 2021W | 0 | 0 | 0 | 0 | 1 | Analysis of the current status of cognitive weakness and its influencing factors in elderly Hemodialysis patients |
| Wang 2022W | 0 | 0 | 0 | 0 | 1 | Current status of cognitive frailty in elderly maintenance Hemodialysis patients and exploration of its main influencing factors |
| Wei 2024 | 0 | 0 | 0 | 0 | 1 | Construction and application of a risk predic tion model of cognitive frailty in maintenance Hemodialysis patients. |
| Xu 2023 | 0 | 0 | 0 | 0 | 1 | Analysis of the influencing factors of frailty in uremic maintenance Hemodialysis patients and their effects on cognitive function  and Micro inflammatory state |
| Yao 2024 | 0 | 0 | 0 | 0 | 1 | Analysis of cognitive frailty and its influencing factors in  elderly Hemodialysis patients |
| Yi 2024 | 0 | 0 | 0 | 0 | 1 | Influencing factors and interventional measures for cognitive frailty in patients on maintenance Hemodialysis |
| Yuan 2024 | 0 | 0 | 0 | 0 | 1 | Analysis of influencing factors of physical frailty complicated with cognitive impairment in elderly patients with maintenance Hemodialysis |
| Fadili 2014 | 1 | 0 | 1 | 1 | 0 | Prevalence and risk factors of cognitive dysfunction in chronic hemodialysis patients. |
| McAdams 2015 | 0 | 1 | 0 | 1 | 0 | Frailty and cognitive function in incident hemodialysis patients |
| Odagiri 2011 | 1 | 0 | 0 | 1 | 0 | Cognitive function among hemodialysis patients in Japan |
| Afsar & Elsurer 2015 | 0 | 0 | 0 | 1 | 0 | Association between serum bicarbonate and pH with depression, cognition and sleep quality in hemodialysis patients |
| Afsar 2013 | 0 | 0 | 0 | 1 | 0 | The relation between Internet and social media use and the demographic and clinical parameters, quality of life, depression, cognitive function and sleep quality in hemodialysis patients: social media and hemodialysis |
| Agnanis 2010 | 0 | 0 | 0 | 1 | 0 | Depression and cognitive function in maintenance hemodialysis patients |
| Bossola 2011 | 0 | 0 | 0 | 1 | 0 | Mini Mental State Examination over time in chronic hemodialysis patients |
| Bossola 2012 | 0 | 0 | 0 | 1 | 0 | Relationship between appetite and symptoms of depression and anxiety in patients  on chronic hemodialysis |
| Bossola 2013 | 0 | 0 | 0 | 1 | 0 | Qualities of fatigue in patients on chronic hemodialysis |
| Bossola 2014 | 0 | 0 | 0 | 1 | 0 | Cognitive performance is associated with left ventricular function in older chronic  hemodialysis patients: result of a pilot study |
| Gad 2012 | 0 | 0 | 0 | 1 | 0 | Cognitive impairment in hemodialysis patients |
| Giang 2011 | 0 | 0 | 0 | 1 | 0 | Cognitive function and dialysis adequacy: No clear relationship |
| Giang 2013 | 0 | 0 | 0 | 1 | 0 | Measures of blood pressure and cognition in dialysis patients |
| Hain 2008 | 0 | 0 | 0 | 1 | 0 | Cognitive function and adherence of older adults undergoing hemodialysis |
| Jang 2012 | 0 | 0 | 0 | 1 | 0 | Self-care behaviors and physiological index according to cognitive function of hemodialysis patients |
| Jassal 2008 | 0 | 0 | 0 | 1 | 0 | Differential impairment of psychomotor efficiency and processing speed in patients with chronic kidney disease |
| Jung 2013 | 0 | 0 | 0 | 1 | 0 | Relationship between cognitive impairment and depression in dialysis patients |
| Kalaitzidis 2013 | 0 | 0 | 0 | 1 | 0 | Risk factors for cognitive dysfunction in CKD and hypertensive subjects |
| Kavanagh 2015 | 0 | 0 | 0 | 1 | 0 | Prevalence and correlates of functional dependence among maintenance dialysis patients |
| Kitaguchi 2015 | 0 | 0 | 0 | 1 | 0 | A prospective study on blood Abeta levels and the cognitive function of patients  with hemodialysis: A potential therapeutic strategy for Alzheimer's disease |
| Kurella Tamura 2010 | 0 | 0 | 0 | 1 | 0 | The Frequent Hemodialysis Network Trial Group |
| Kurella Tamura 2012 | 0 | 0 | 0 | 1 | 0 | Prevalence and significance of stroke symptoms among patients receiving  maintenance dialysis |
| Leinau 2009 | 0 | 0 | 0 | 1 | 0 | Relationship between conditions addressed by hemodialysis guidelines and non-ESRD-specific conditions affecting quality of life |
| Murray 2006 | 0 | 0 | 0 | 1 | 0 | Cognitive impairment in hemodialysis patients is common |
| Okpechi 2013 | 0 | 0 | 0 | 1 | 0 | Health-related quality of life in patients on hemodialysis and peritoneal dialysis |
| Ozcan 2015 | 0 | 0 | 0 | 1 | 0 | Kidney transplantation Is superior to hemodialysis and peritoneal dialysis in terms of cognitive function, anxiety and depression symptoms in chronic kidney disease |
| Paramanantham 2011 | 0 | 0 | 0 | 1 | 0 | Adverse effects in hospice patients with chronic kidney disease receiving hydromorphone |
| Rodriguez 2013 | 0 | 0 | 0 | 1 | 0 | Association of sleep disturbances with cognitive impairment and depression in  maintenance hemodialysis patients |
| Seidel 2013 | 0 | 0 | 0 | 1 | 0 | The prevalence, severity and association with HbA1c and fibrinogen of cognitive impairment in chronic kidney disease |
| Seidel 2014 | 0 | 0 | 0 | 1 | 0 | Physical, cognitive and emotional factors contributing to quality of life,  functional health and participation in community dwelling in chronic kidney disease |
| Shaffi 2013 | 0 | 0 | 0 | 1 | 0 | Low 25-hydroxyvitamin D levels and cognitive impairment in hemodialysis patients |
| Shavit 2014 | 0 | 0 | 0 | 1 | 0 | Mild hyponatremia is associated with functional and cognitive decline in chronic hemodialysis patients |
| Sorensen 2012 | 0 | 0 | 0 | 1 | 0 | The kidney disease quality of life cognitive function subscale and  cognitive performance in maintenance hemodialysis patients |
| Tholen 2014 | 0 | 0 | 0 | 1 | 0 | Variability of cognitive performance during hemodialysis: standardization of cognitive assessment |
| Tholen 2016 | 0 | 0 | 0 | 1 | 0 | Reduction of amyloid-beta plasma levels by hemodialysis: An anti amyloid treatment strategy? |
| Tiffin 2014 | 0 | 0 | 0 | 1 | 0 | The Montreal Cognitive Assessment (MoCA)-A sensitive screening instrument for detecting cognitive impairment in chronic hemodialysis patients |
| Vazquez 2005 | 0 | 0 | 0 | 1 | 0 | Spanish Cooperative Renal Patients Quality of Life Study Group |
| Wolfgram 2014 | 0 | 0 | 0 | 1 | 0 | Haemodynamics during dialysis and cognitive performance |
| Zabet 2014 | 0 | 0 | 0 | 1 | 0 | Depression in patients with chronic kidney disease on dialysis in Saudi Arabia |
| Lu 2019 | 1 | 0 | 1 | 0 | 0 | The incidence prognosis and risk factors of cog nitive impairment in maintenance Haemodialysis patients. |
| Feng 2021 | 0 | 0 | 1 | 0 | 0 | The assessment of cognitive impairment in maintenance hemodialysis patients and the relationship between cognitive impairment and depressive symptoms |
| Karakizlis 2022 | 0 | 0 | 1 | 0 | 0 | Assessment of cognitive impairment and related risk factors in hemodialysis patients |
| Luo 2020 | 0 | 0 | 1 | 0 | 0 | Cognitive impairment and associated risk factors in older adult hemodialysis patients: a cross-sectional survey |
| Ou 2022 | 0 | 0 | 1 | 0 | 0 | Study on Correlation between Body Cell Mass Index and Cognitive Impairment in Hemodialysis Maintaining Patients: A Cross-Sectional Study |
| Tian 2022 | 0 | 0 | 1 | 0 | 0 | Cognitive dysfunction and its risk factors in patients undergoing maintenance emodialysis |
| Yang 2022 | 0 | 0 | 1 | 0 | 0 | The Impact of Malnutrition, Inflammation on Cognitive Impairment in Hemodialysis Patients: A Multicenter Study |
| Zhao 2022 | 0 | 0 | 1 | 0 | 0 | Relationship between physical perfor mance and mild cognitive impairment in elderly hemodialysis patients is modified by the presence of diabetes: a multicenter cross-sectional study |
| Zubair 2017 | 0 | 0 | 1 | 0 | 0 | Association Of Quality Of Sleep With Cognitive Decline Among The Patients Of Chronic Kidney Disease Undergoing Haemodialysis |
| Chen 2021a | 0 | 1 | 0 | 0 | 0 | Correlation Between Cognitive Dysfunction and Frailty in Maintenance Hemodialysis Patients |
| Chen 2021b | 0 | 1 | 0 | 0 | 0 | Current Status and Influenc ing Factors of Cognitive Frailty in Maintenance Hemodialysis Patients |
| Chen 2022 | 0 | 1 | 0 | 0 | 0 | Com parison of the Prevalence and Associated Factors of Cognitive Frailty  Between Elderly and Middle-Young Patients Receiving Maintenance  Hemodialysis |
| Gopinathan 2020 | 0 | 1 | 0 | 0 | 0 | The Prevalence of Frailty and Its Association With Cognitive Dysfunction Among Elderly Patients on Maintenance Hemodialysis: A Cross-Sectional Study From South India |
| Jafari 2020 | 0 | 1 | 0 | 0 | 0 | The Bur den of Frailty on Mood, Cognition, Quality of Life, and Level of Inde pendence in Patients on Hemodialysis: Regina Hemodialysis Frailty study |
| Li 2023C | 0 | 1 | 0 | 0 | 0 | Establishment of Cognitive Frailty Risk Prediction Model in Elderly Patients With Maintenance Hemodialysis |
| Liu 2023Ca | 0 | 1 | 0 | 0 | 0 | Construction of Cognitive Frailty Risk Prediction Model in Elderly Maintenance Hemodialysis Patients |
| Liu 2023Cb | 0 | 1 | 0 | 0 | 0 | Investigation and Analysis of Cognitive Frailty and Its Influencing Factors in Maintenance Hemodi alysis Patients in Nanjing |
| Wang 2019 | 0 | 1 | 0 | 0 | 0 | Study on Somatic and Cog nitive Frailties and Their Influencing Factors in Elderly Hospitalized  Patients |
| Wang 2021C | 0 | 1 | 0 | 0 | 0 | Analysis of Cognitive Frailty and Its Influencing Factors in Elderly Hemodialysis Patients |
| Wang 2022C | 0 | 1 | 0 | 0 | 0 | Status of Cognitive Frailty in Elderly Maintenance Hemodialysis Patients and Its Main Influencing  Factors |
| Cai 2019 | 1 | 0 | 0 | 0 | 0 | Relationship between plasma T-tau, Aβ-42 protein content and mild cognitive impairment in dialysis patients |
| Chen 2012 | 1 | 0 | 0 | 0 | 0 | The prevalence of cognitive impairment and its risk fac tors in patients with hemodialysis |
| Chen 2021C | 1 | 0 | 0 | 0 | 0 | Relationshp between cognitive impairment and frailty in mainte nance hemodialysis patients |
| Cho 2019 | 1 | 0 | 0 | 0 | 0 | Association of intracranial artery calcification with cognitive impairment in hemodialysis patients |
| Chou 2015 | 1 | 0 | 0 | 0 | 0 | Longer dialysis times are associated with a lower chance of dementia among chronic hemodialysis patients |
| Guo 2018 | 1 | 0 | 0 | 0 | 0 | Association between cognitive impairments and blood pressure variability during the day/night cycle in elderly patients treated with hemodialysis |
| Guo 2019 | 1 | 0 | 0 | 0 | 0 | Clinical investigation of the association between serum homocysteine and cognitive impairment in elderly patients with hemodialysis |
| Guo 2021 | 1 | 0 | 0 | 0 | 0 | A cross-sectional study on the char acteristics of cognitive impairment in middle-aged and elderly patients undergoing maintenance hemodialysis |
| He 2021 | 1 | 0 | 0 | 0 | 0 | Analysis of related factors and TCM syndrome character istics of mild cognitive impairment in maintenance hemodialysis pa tients |
| Hou 2019 | 1 | 0 | 0 | 0 | 0 | Analysis of cognitive impair ment and related risk factors in maintenance hemodialysis patients with end stage renal disease |
| Huang 2021 | 1 | 0 | 0 | 0 | 0 | The correlation between mild cognitive impairment and hypotension in young and middle aged maintenance hemodialysis patients |
| Kim 2017 | 1 | 0 | 0 | 0 | 0 | Association of arterial stiffness and central pressure with cognitive function in incident hemodi alysis patients: The PACE study |
| Lee 2020 | 1 | 0 | 0 | 0 | 0 | Association of nutritional status with osteoporosis, sarcope nia, and cognitive impairment in patients on hemodialysis |
| Li 2021 | 1 | 0 | 0 | 0 | 0 | Changes of serum homocysteine, cysta tin C and hypersensitive C-reactive protein levels in patients with maintenance hemodialysis and their correlation with cognitive dysfunction |
| Lin 2019 | 1 | 0 | 0 | 0 | 0 | Indole-3 acetic acid in creased risk of impaired cognitive function in patients receiving he modialysis |
| Liu 2016 | 1 | 0 | 0 | 0 | 0 | Risk factors of cognitive impairment in patients with mainte nance hemodialysis and characteristics of TCM syndromes |
| Nakai 2018 | 1 | 0 | 0 | 0 | 0 | Is hemodialysis itself a risk factor for dementia? An analysis  of nationwide registry data of patients on maintenance hemodi alysis in Japan |
| Pang 2021 | 1 | 0 | 0 | 0 | 0 | Association of residual renal function with cognitive function among maintenance hemodialysis patients. |
| Park 2020 | 1 | 0 | 0 | 0 | 0 | Serum S100B represents a bio marker for cognitive impairment in patients with end-stage renal disease |
| Shui 2021 | 1 | 0 | 0 | 0 | 0 | Association between platelet/lymphocyte ratio and neutro phils/lymphocyte ratio with cognitive impairment in maintenance he modialysis patients |
| Song 2019 | 1 | 0 | 0 | 0 | 0 | Prediction of cog nitive impairment in hemodialysis patients |
| Tamura 2010 | 1 | 0 | 0 | 0 | 0 | Prevalence and correlates of cognitive impairment in hemodialysis patients: The frequent  hemodialysis network trials |
| Tao 2021 | 1 | 0 | 0 | 0 | 0 | Analysis on change characteristics and influencing factors of cognitive function in perimenopausal patients treated by maintenance hemodialysis |
| Tian 2020 | 1 | 0 | 0 | 0 | 0 | Correlation between cerebral he modynamic changes and cognitive impairment in hemodialysis pa tients |
| Tian 2021 | 1 | 0 | 0 | 0 | 0 | Effects of sleep quality on cognitive function in elderly patients undergoing hemodialysis |
| Wu 2021a | 1 | 0 | 0 | 0 | 0 | Characteristics of cognitive dys function in patients with maintenance hemodialysis and its rela tionship with prognosis |
| Wu Qin Zhang 2021 | 1 | 0 | 0 | 0 | 0 | Relationship between body composi tion and cognitive impairment in patients with maintenance hemo dialysis |
| Xiao 2021 | 1 | 0 | 0 | 0 | 0 | Correlation between home systolic blood pressure vari ability and cognitive impairment in maintenance hemodialysis pa tients |
| Zhang 2021 | 1 | 0 | 0 | 0 | 0 | Cognitive assessment tools for mild cognitive impairment screening |
| Zhao 2020 | 1 | 0 | 0 | 0 | 0 | Analysis of related fac tors affecting cognitive function in hemodialysis patients |
| Zhao 2021 | 1 | 0 | 0 | 0 | 0 | Investigation of cognitive impairment and left ventricular ejection fraction in maintenance hemodialysis patients [Master's the sis, Shanxi Medical University] |
| Zhong 2018 | 1 | 0 | 0 | 0 | 0 | Risk factors of cognitive dys function in elderly patients undergoing maintenance hemodialysis |
| Zhou 2020 | 1 | 0 | 0 | 0 | 0 | Occurrence of cognitive im pairment and analysis of influential factors in elderly patients with maintenance hemodialysis |
| Zhu 2018 | 1 | 0 | 0 | 0 | 0 | Risk factors analysis of cognitive dysfunction in hemodial ysis patients and construction of prediction model |
