# Supplementary material for: Risk factors for cognitive impairment in patients undergoing hemodialysis: an umbrella review and meta-analysis
Source: Ren Fail. 2026 Nov 3;48(1):2741985. doi: 10.1080/0886022X.2026.2741985 (PMC13757910; doi:10.1080/0886022X.2026.2741985)
Supplement: Supplementary file 4 [file IRNF_A_2741985_SM8707.docx]

| Tale S4 GRADE rating | | | | | | | | | | | | | | | | |
| --- | --- | --- | --- | --- | --- | --- | --- | --- | --- | --- | --- | --- | --- | --- | --- | --- |
| **Study** | **Risk Factors** | **Control** | **Outcomes** | **No. of studies** | **RCT** | **Cohort** | **case-control/cross-sectional/other** | **Risk of Bias** | **Inconsistency** | **Indirectness** | **Imprecision** | **Publication bias** | **Plausible confounding** | **Magnitude of** | **Dose-response gradient** | **Quality** |
|  |  |  |  |  |  |  |  |  |  |  |  |  |  | **effect** |  |  |
| Cao 2022 | every year increase | NA | risk factor | 20 | 0 | 0 | 20 | no serious | serious | no serious | serious | serious | no serious | no | no | very low |
| Cao 2022 | every 10-year increase | NA | risk factor | 4 | 0 | 0 | 3 | no serious | serious | no serious | no serious | serious | no serious | no | no | very low |
| Cao 2022 | older age | <18 years old | risk factor | 27 | 0 | 0 | 26 | no serious | serious | no serious | no serious | serious | no serious | no | no | very low |
| Cheng 2024 | Age | Young | risk factor | 9 | 0 | 0 | 9 | no serious | serious | no serious | serious | no serious | no serious | no | no | very low |
| Liu 2023 | Age | Young | risk factor | 9 | 0 | 0 | NA | no serious | serious | no serious | serious | no serious | no serious | no | no | very low |
| Oh 2019 | Age | NA | risk factor | 7 | 0 | NA | NA | no serious | no serious | no serious | serious | no serious | no serious | no | no | very low |
| Wei 2025 | Age >60 | Age≤60 | risk factor | 2 | 0 | NA | NA | no serious | no serious | no serious | serious | no serious | no serious | no | no | very low |
| Wei 2025 | Age >75 | Age≤75 | risk factor | 5 | 0 | NA | NA | no serious | no serious | no serious | serious | no serious | no serious | no | no | very low |
| Cao 2022 | Female sex | male | risk factor | 8 | 0 | 0 | 7 | no serious | serious | no serious | no serious | no serious | no serious | no | no | very low |
| Liu 2023 | Female |  | risk factor | 10 | 0 | 0 | NA | no serious | serious | no serious | serious | no serious | no serious | no | no | very low |
| Oh 2019 | Gender | NA | risk factor | 9 | 0 | NA | NA | no serious | serious | no serious | serious | no serious | no serious | no | no | very low |
| Wei 2025 | Female | male | risk factor | 2 | 0 | NA | NA | no serious | no serious | no serious | serious | no serious | no serious | yes | no | low |
| Cao 2022 | Educational level | without | risk factor | 19 | 0 | 0 | 19 | no serious | serious | no serious | serious | serious | no serious | no | no | very low |
| Cao 2022 | Education (every year increase) | NA | risk factor | 11 | 0 | 0 | 11 | no serious | serious | no serious | serious | serious | no serious | no | no | very low |
| Cao 2022 | Education (≥12 years) | <12 years | risk factor | 7 | 0 | 0 | 7 | no serious | serious | no serious | serious | serious | no serious | yes | no | very low |
| Cheng 2024 | Educational level | High | risk factor | 3 | 0 | 0 | 3 | no serious | serious | no serious | serious | no serious | no serious | yes | no | very low |
| Oh 2019 | Education | NA | risk factor | 9 | 0 | NA | NA | no serious | serious | no serious | serious | no serious | no serious | no | no | very low |
| Wei 2025 | Educational Level | NA | risk factor | 2 | 0 | NA | NA | no serious | no serious | no serious | serious | no serious | no serious | yes | no | low |
| Cao 2022 | Primary language not English | English | risk factor | 1 | 0 | 0 | 1 | no serious | undected | no serious | serious | undected | no serious | yes | no | low |
| Cao 2022 | Place of residence: hospitals | Other places | risk factor | 1 | 0 | 0 | 0 | no serious | undected | no serious | no serious | undected | no serious | yes | no | moderate |
| Cheng 2024 | Social support | without | risk factor | 2 | 0 | 0 | 2 | no serious | serious | no serious | serious | no serious | no serious | no | no | very low |
| Cao 2022 | Body mass index | NA | risk factor | 3 | 0 | 0 | 2 | no serious | serious | no serious | no serious | no serious | no serious | no | no | very low |
| Cao 2022 | Sleep quality | good sleep quality | risk factor | 3 | 0 | 0 | 3 | no serious | serious | no serious | serious | no serious | no serious | yes | no | very low |
| Oh 2019 | Sleep difficulties | NA | risk factor | 3 | 0 | NA | NA | no serious | no serious | no serious | serious | no serious | no serious | no | no | very low |
| Cheng 2024 | Waistline | NA | risk factor | 2 | 0 | 0 | 2 | no serious | no serious | no serious | serious | no serious | no serious | no | no | very low |
| Wei 2025 | Waist | NA | risk factor | 2 | 0 | NA | NA | no serious | no serious | no serious | serious | no serious | no serious | no | no | very low |
| Wei 2025 | Calf circumference | NA | risk factor | 2 | 0 | NA | NA | no serious | no serious | no serious | serious | no serious | no serious | no | no | very low |
| Liu 2023 | Smoking use | without | risk factor | 5 | 0 | 0 | NA | no serious | serious | no serious | serious | no serious | no serious | no | no | very low |
| Liu 2023 | Alcohol use | without | risk factor | 3 | 0 | 0 | NA | no serious | no serious | no serious | serious | no serious | no serious | no | no | very low |
| Wei 2025 | Exercise | NA | risk factor | 2 | 0 | NA | NA | no serious | serious | no serious | serious | no serious | no serious | no | no | very low |
| Cao 2022 | Slow gait | NA | risk factor | 1 | 0 | 0 | 1 | no serious | undected | no serious | serious | undected | no serious | yes | no | low |
| Cao 2022 | Low grip strength | NA | risk factor | 1 | 0 | 0 | 1 | no serious | undected | no serious | serious | undected | no serious | yes | no | low |
| Cao 2022 | Activities of daily living | NA | risk factor | 1 | 0 | 0 | 1 | no serious | undected | no serious | no serious | undected | no serious | no | no | low |
| Oh 2019 | ADL | NA | risk factor | 3 | 0 | NA | NA | no serious | no serious | no serious | serious | no serious | no serious | no | no | very low |
| Oh 2019 | Pain | NA | risk factor | 3 | 0 | NA | NA | no serious | no serious | no serious | serious | no serious | no serious | no | no | very low |
| Cao 2022 | History of limb amputation | NA | risk factor | 1 | 0 | 0 | 1 | no serious | undected | no serious | no serious | undected | no serious | no | no | low |
| Cao 2022 | History of hip fracture | NA | risk factor | 1 | 0 | 0 | 1 | no serious | undected | no serious | no serious | undected | no serious | yes | no | moderate |
| Wei 2025 | Fall history | without | risk factor | 2 | 0 | NA | NA | no serious | no serious | no serious | serious | no serious | no serious | yes | no | low |
| Cao 2022 | Depression status | normal | risk factor | 1 | 0 | 0 | 1 | no serious | undected | no serious | serious | undected | no serious | no | no | very low |
| Cheng 2024 | Depression | without | risk factor | 6 | 0 | 0 | 6 | no serious | serious | no serious | serious | no serious | no serious | yes | no | very low |
| Oh 2019 | Depression | NA | risk factor | 9 | 0 | NA | NA | no serious | serious | no serious | serious | no serious | no serious | no | no | very low |
| Wei 2025 | Depression(HADS) | without | risk factor | 3 | 0 | NA | NA | no serious | no serious | no serious | serious | no serious | no serious | yes | no | low |
| Wei 2025 | Depression | without | risk factor | 3 | 0 | NA | NA | no serious | serious | no serious | serious | no serious | no serious | no | no | very low |
| Wei 2025 | Depression(SDS) | without | risk factor | 2 | 0 | NA | NA | no serious | serious | no serious | serious | no serious | no serious | no | no | very low |
| Wei 2025 | Geriatric Depression Scale score(GDS-15) | NA | risk factor | 2 | 0 | NA | NA | no serious | no serious | no serious | serious | no serious | no serious | no | no | very low |
| Cao 2022 | Hope level | NA | risk factor | 1 | 0 | 0 | 1 | no serious | undected | no serious | serious | undected | no serious | yes | no | low |
| Cao 2022 | Psychological status | NA | risk factor | 1 | 0 | 0 | 1 | no serious | undected | no serious | serious | undected | no serious | yes | no | low |
| Cao 2022 | Hypertension | without | risk factor | 4 | 0 | 0 | 4 | no serious | serious | no serious | serious | no serious | no serious | yes | no | very low |
| Liu 2023 | Hypertension | without | risk factor | 6 | 0 | 0 | NA | no serious | no serious | no serious | serious | no serious | no serious | no | no | very low |
| Oh 2019 | Hypertension | NA | risk factor | 7 | 0 | NA | NA | no serious | serious | no serious | serious | no serious | no serious | no | no | very low |
| Cao 2022 | Diabetes | without | risk factor | 14 | 0 | 0 | 13 | no serious | serious | no serious | no serious | serious | no serious | yes | no | very low |
| Liu 2023 | Diabetes | without | risk factor | 7 | 0 | 0 | NA | no serious | serious | no serious | serious | no serious | no serious | no | no | very low |
| Oh 2019 | Diabetes | NA | risk factor | 7 | 0 | NA | NA | no serious | serious | no serious | serious | no serious | no serious | no | no | very low |
| Cao 2022 | Heart disease | without | risk factor | 5 | 0 | 0 | 4 | no serious | serious | no serious | no serious | no serious | no serious | no | no | very low |
| Liu 2023 | Coronary heart disease | without | risk factor | 2 | 0 | 0 | NA | no serious | serious | no serious | serious | no serious | no serious | no | no | very low |
| Oh 2019 | CVD | NA | risk factor | 9 | 0 | NA | NA | no serious | serious | no serious | serious | no serious | no serious | no | no | very low |
| Cao 2022 | Cerebrovascular accident | without | risk factor | 4 | 0 | 0 | 4 | no serious | no serious | no serious | serious | no serious | no serious | no | no | very low |
| Liu 2023 | Cerebrovascular disease | without | risk factor | 4 | 0 | 0 | NA | no serious | no serious | no serious | serious | no serious | no serious | no | no | very low |
| Cheng 2024 | Stroke history | without | risk factor | 3 | 0 | 0 | 3 | no serious | no serious | no serious | serious | no serious | no serious | yes | no | low |
| Liu 2023 | Stroke | without | risk factor | 3 | 0 | 0 | NA | no serious | no serious | no serious | serious | no serious | no serious | no | no | very low |
| Oh 2019 | Stroke | NA | risk factor | 4 | 0 | NA | NA | no serious | no serious | no serious | serious | no serious | no serious | no | no | very low |
| Wei 2025 | Stroke history | without | risk factor | 2 | 0 | NA | NA | no serious | no serious | no serious | serious | no serious | no serious | yes | no | low |
| Cao 2022 | SBPV | NA | risk factor | 2 | 0 | 0 | 2 | no serious | no serious | no serious | serious | no serious | no serious | no | no | very low |
| Cao 2022 | Mean systolic blood pressure | NA | risk factor | 1 | 0 | 0 | 1 | no serious | undected | no serious | serious | undected | no serious | no | no | very low |
| Cao 2022 | Arterial stiffness | NA | risk factor | 2 | 0 | 0 | 2 | no serious | no serious | no serious | serious | no serious | no serious | no | no | very low |
| Cao 2022 | Multiple comorbid conditions | without | risk factor | 3 | 0 | 0 | 3 | no serious | no serious | no serious | serious | no serious | no serious | yes | no | low |
| Wei 2025 | Comorbidities | without | risk factor | 2 | 0 | NA | NA | no serious | no serious | no serious | serious | no serious | no serious | yes | no | low |
| Wei 2025 | Complications | without | risk factor | 2 | 0 | NA | NA | no serious | no serious | no serious | serious | no serious | no serious | yes | no | low |
| Cheng 2024 | Complication | without | risk factor | 6 | 0 | 0 | 6 | no serious | no serious | no serious | serious | no serious | no serious | no | no | very low |
| Wei 2025 | CCI | NA | risk factor | 2 | 0 | NA | NA | no serious | no serious | no serious | serious | no serious | no serious | yes | no | low |
| Cao 2022 | Haemodialysis duration | shorter | risk factor | 13 | 0 | 0 | 12 | no serious | no serious | no serious | no serious | serious | no serious | no | no | very low |
| Cheng 2024 | Dialysis duration | NA | risk factor | 4 | 0 | 0 | 4 | no serious | no serious | no serious | serious | no serious | no serious | no | no | very low |
| Oh 2019 | Duration | NA | risk factor | 7 | 0 | NA | NA | no serious | no serious | no serious | serious | no serious | no serious | no | no | very low |
| Liu 2023 | Time on dialysis | NA | risk factor | 6 | 0 | 0 | NA | no serious | no serious | no serious | serious | no serious | no serious | no | no | very low |
| Cao 2022 | Haemodialysis adequacy | Insufficient | risk factor | 7 | 0 | 0 | 6 | no serious | no serious | no serious | no serious | no serious | no serious | no | no | low |
| Cao 2022 | Haemodialysis frequency | NA | risk factor | 2 | 0 | 0 | 2 | no serious | no serious | no serious | serious | no serious | no serious | no | no | very low |
| Oh 2019 | Effects | NA | risk factor | 9 | 0 | NA | NA | no serious | no serious | no serious | serious | no serious | no serious | no | no | very low |
| Wei 2025 | Dialysis age | NA | risk factor | 2 | 0 | NA | NA | no serious | no serious | no serious | serious | no serious | no serious | yes | no | low |
| Cao 2022 | Post-dialysis diastolic pressure | NA | risk factor | 1 | 0 | 0 | 1 | no serious | undected | no serious | serious | undected | no serious | yes | no | low |
| Cao 2022 | Excessive body weight changes before and after dialysis | normal | risk factor | 1 | 0 | 0 | 1 | no serious | undected | no serious | serious | undected | no serious | yes | no | low |
| Cao 2022 | Interdialysis hypotension | normal | risk factor | 1 | 0 | 0 | 1 | no serious | undected | no serious | serious | undected | no serious | yes | no | low |
| Cao 2022 | Nocturnal haemodialysis | other time | risk factor | 1 | 0 | 0 | 1 | no serious | undected | no serious | serious | undected | no serious | yes | no | low |
| Cao 2022 | Low-flux haemodialysis | normal | risk factor | 1 | 0 | 0 | 1 | no serious | undected | no serious | serious | undected | no serious | yes | no | low |
| Cao 2022 | Dry weight | NA | risk factor | 2 | 0 | 0 | 2 | no serious | no serious | no serious | serious | no serious | no serious | yes | no | low |
| Cao 2022 | Creatinine | NA | risk factor | 4 | 0 | 0 | 4 | no serious | no serious | no serious | serious | no serious | no serious | no | no | very low |
| Oh 2019 | Creatinine | NA | risk factor | 3 | 0 | NA | NA | no serious | no serious | no serious | serious | no serious | no serious | no | no | very low |
| Cheng 2024 | Serum creatinine | NA | risk factor | 3 | 0 | 0 | 3 | no serious | no serious | no serious | serious | no serious | no serious | no | no | very low |
| Wei 2025 | Serum creatinine level | NA | risk factor | 3 | 0 | NA | NA | no serious | no serious | no serious | serious | no serious | no serious | no | no | very low |
| Oh 2019 | BUN | NA | risk factor | 4 | 0 | NA | NA | no serious | no serious | no serious | serious | no serious | no serious | no | no | very low |
| Cao 2022 | Cystatin C | NA | risk factor | 1 | 0 | 0 | 1 | no serious | undected | no serious | serious | undected | no serious | yes | no | low |
| Cao 2022 | Residual urine volume | NA | risk factor | 1 | 0 | 0 | 1 | no serious | undected | no serious | serious | undected | no serious | yes | no | low |
| Cao 2022 | Chronic glomerulonephritis | NA | risk factor | 1 | 0 | 0 | 1 | no serious | undected | no serious | serious | undected | no serious | no | no | very low |
| Cao 2022 | Haemoglobin level | high | risk factor | 14 | 0 | 0 | 13 | no serious | no serious | no serious | no serious | serious | no serious | no | no | very low |
| Cheng 2024 | Hemoglobin | NA | risk factor | 3 | 0 | 0 | 3 | no serious | no serious | no serious | serious | no serious | no serious | no | no | very low |
| Oh 2019 | Hemoglobin | NA | risk factor | 8 | 0 | NA | NA | no serious | no serious | no serious | serious | no serious | no serious | no | no | very low |
| Wei 2025 | Haemoglobin | NA | risk factor | 3 | 0 | NA | NA | no serious | no serious | no serious | serious | no serious | no serious | no | no | very low |
| Cao 2022 | Serum albumin level |  | risk factor | 5 | 0 | 0 | 3 | no serious | no serious | no serious | no serious | no serious | no serious | no | no | low |
| Cheng 2024 | Serum albumin | NA | risk factor | 4 | 0 | 0 | 4 | no serious | no serious | no serious | serious | no serious | no serious | no | no | very low |
| Liu 2023 | Albumin | NA | risk factor | 3 | 0 | 0 | NA | no serious | no serious | no serious | serious | no serious | no serious | no | no | very low |
| Oh 2019 | Albumin | NA | risk factor | 6 | 0 | NA | NA | no serious | no serious | no serious | serious | no serious | no serious | no | no | very low |
| Wei 2025 | Serum albumin | NA | risk factor | 6 | 0 | NA | NA | no serious | no serious | no serious | serious | no serious | no serious | no | no | very low |
| Cao 2022 | 25(OH)D | NA | risk factor | 2 | 0 | 0 | 2 | no serious | no serious | no serious | serious | no serious | no serious | no | no | very low |
| Cao 2022 | CRP level | NA | risk factor | 2 | 0 | 0 | 1 | no serious | no serious | no serious | no serious | no serious | no serious | no | no | low |
| Cao 2022 | Homocysteine | NA | risk factor | 2 | 0 | NA | NA | no serious | no serious | no serious | serious | no serious | no serious | no | no | very low |
| Cao 2022 | Serum cholesterol | NA | risk factor | 2 | 0 | 0 | 2 | no serious | no serious | no serious | serious | no serious | no serious | no | no | very low |
| Cao 2022 | Serum uric acid | NA | risk factor | 4 | 0 | 0 | 4 | no serious | no serious | no serious | serious | no serious | no serious | yes | no | low |
| Cao 2022 | PTH | NA | risk factor | 6 | 0 | 0 | 4 | no serious | no serious | no serious | serious | no serious | no serious | no | no | very low |
| Cao 2022 | Sodium | NA | risk factor | 1 | 0 | 0 | 1 | no serious | undected | no serious | serious | undected | no serious | no | no | very low |
| Cao 2022 | Potassium | NA | risk factor | 1 | 0 | 0 | 1 | no serious | undected | no serious | serious | undected | no serious | no | no | very low |
| Cao 2022 | Phosphorus | NA | risk factor | 1 | 0 | 0 | 1 | no serious | undected | no serious | serious | undected | no serious | no | no | very low |
| Cao 2022 | Blood urea nitrogen | NA | risk factor | 1 | 0 | NA | NA | no serious | undected | no serious | serious | undected | no serious | no | no | very low |
| Cheng 2024 | Malnutrition | normal | risk factor | 3 | 0 | 0 | 3 | no serious | no serious | no serious | serious | no serious | no serious | yes | no | low |
| Wei 2025 | Malnutrition | NA | risk factor | 3 | 0 | NA | NA | no serious | no serious | no serious | serious | no serious | no serious | yes | no | low |
| Wei 2025 | Triglycerides | NA | risk factor | 2 | 0 | NA | NA | no serious | no serious | no serious | serious | no serious | no serious | yes | no | low |
| Cao 2022 | Erythrocyte count | NA | risk factor | 1 | 0 | 0 | 1 | no serious | undected | no serious | serious | undected | no serious | no | no | very low |
| Cao 2022 | Fibroblast growth factor-23 | NA | risk factor | 1 | 0 | 0 | 1 | no serious | undected | no serious | serious | undected | no serious | no | no | very low |
| Cao 2022 | Plasma T-tau | NA | risk factor | 1 | 0 | 0 | 1 | no serious | undected | no serious | serious | undected | no serious | no | no | very low |
| Cao 2022 | Neuron-specific enolase | NA | risk factor | 1 | 0 | 0 | 1 | no serious | undected | no serious | serious | undected | no serious | yes | no | low |
| Cao 2022 | S100B | NA | risk factor | 1 | 0 | 0 | 1 | no serious | undected | no serious | serious | undected | no serious | yes | no | low |
| Cao 2022 | Intracranial artery calcification | NA | risk factor | 1 | 0 | 0 | 1 | no serious | undected | no serious | serious | undected | no serious | yes | no | low |
| Cao 2022 | Lean tissue mass | NA | risk factor | 1 | 0 | 0 | 1 | no serious | undected | no serious | serious | undected | no serious | yes | no | low |
| Cao 2022 | Total body water | NA | risk factor | 1 | 0 | 0 | 1 | no serious | undected | no serious | serious | undected | no serious | no | no | very low |
| Cao 2022 | Extracellular water | NA | risk factor | 1 | 0 | 0 | 1 | no serious | undected | no serious | serious | undected | no serious | yes | no | low |
| Cao 2022 | Neutrophil percentage | NA | risk factor | 1 | 0 | 0 | 1 | no serious | undected | no serious | serious | undected | no serious | no | no | very low |
| Cao 2022 | Platelet/lymphocyte ratio | NA | risk factor | 1 | 0 | 0 | 1 | no serious | undected | no serious | serious | undected | no serious | yes | no | low |
| Cao 2022 | Neutrophils/lymphocyte ratio | NA | risk factor | 1 | 0 | NA | NA | no serious | undected | no serious | serious | undected | no serious | no | no | very low |
